# Species Richness, Molecular Taxonomy and Biogeography of the Radicine Pond Snails (Gastropoda: Lymnaeidae) in the Old World

**DOI:** 10.1038/s41598-018-29451-1

**Published:** 2018-07-25

**Authors:** Olga V. Aksenova, Ivan N. Bolotov, Mikhail Yu. Gofarov, Alexander V. Kondakov, Maxim V. Vinarski, Yulia V. Bespalaya, Yulia S. Kolosova, Dmitry M. Palatov, Svetlana E. Sokolova, Vitaly M. Spitsyn, Alena A. Tomilova, Oksana V. Travina, Ilya V. Vikhrev

**Affiliations:** 1 Northern Arctic Federal University, Arkhangelsk, Russia; 2 Federal Center for Integrated Arctic Research, Russian Academy of Sciences, Arkhangelsk, Russia; 3 Saint Petersburg State University, Saint Petersburg, Russia; 4 Moscow State University, 119992 Moscow, Russia

## Abstract

The radicine pond snails represent a species-rich and widely distributed group, many species of which are key vectors of human and animal trematodoses. Here we clarify the taxonomy, distribution and evolutionary biogeography of the radicine lymnaeids in the Old World based on the most comprehensive multi-locus molecular dataset sampled to date. We show that the subfamily Amphipepleinae is monophyletic and contains at least ten genus-level clades: *Radix* Montfort, 1810, *Ampullaceana* Servain, 1881, *Peregriana* Servain, 1881, *Tibetoradix* Bolotov, Vinarski & Aksenova **gen**. **nov**., *Kamtschaticana* Kruglov & Starobogatov, 1984, *Orientogalba* Kruglov & Starobogatov, 1985, *Cerasina* Kobelt, 1881, *Myxas* G. B. Sowerby I, 1822, *Bullastra* Bergh, 1901, and *Austropeplea* Cotton, 1942. With respect to our phylogeny, species-delimitation model and morphological data, the Old World fauna includes 35 biological species of radicines. Tibet and Eastern Europe harbor the richest faunas, while East Asia and Africa appear to be the most species-poor areas. The radicine clade could have originated near the Cretaceous – Paleocene boundary. The Miocene great lakes in Eurasia seems to be the most important evolutionary hotspots shaping spatial patterns of recent species richness. Finally, we present the first DNA barcode reference library for the reliable molecular identification of species within this group.

## Introduction

### Diversity and economical importance of the radicine pond snails

The pond snails (Lymnaeidae Rafinesque, 1815) represent one of the most diverse and practically important families of freshwater snails, almost cosmopolitan in its distribution^1–3^. Though there is no a commonly accepted system of the family at the subgeneric and generic levels^4^, most workers agree that the taxonomic structure of the Lymnaeidae is rather complicated, with several subfamilies, genera, and subgenera within it^2,4–9^. Some authors prefer to deal with informal taxonomic groups within this family, each of them to cover morphologically and phylogenetically similar groups of taxa. For example, in an early molecular taxonomic work, Remigio & Blair^10^ studied the phylogenetic relationships within the Lymnaeidae with analyzing such informal groups as the ‘radicine’ and ‘stagnicoline’ pond snails (i.e. those grouped around the genera *Radix* Montfort, 1810, and *Stagnicola* Jeffreys, 1830, respectively). Bargues *et al*.^11^ discuss another informal group, the ‘fossarine’ snails (taxa related to the genus *Galba* Schrank, 1803 = *Fossaria* Westerlund, 1885).

Among these groups, that of radicine snails is restricted almost totally to the Old World in its distribution, with one species, *Radix auricularia* (L., 1758), introduced into North America, Australia and New Zealand^12,13^, and with a few taxa native to Australasia^7,14^. It includes several genera (or subgenera) characterized by a shell with relatively short spire, more or less inflated body whorl and low whorls number (typically 3–4). Apart from *Radix*, four (sub-)genera are usually discussed as being placed to this group: *Cerasina* Kobelt, 1881, *Myxas* Sowerby, 1822, *Bullastra* Bergh, 1901, and *Austropeplea* Cotton, 1942^2,4–6,14,15^. All radicines are caryologically similar, each species having the same haploid chromosome number n = 17^16^.

The radicine snails are known as intermediate hosts of numerous species of flatworms causing health injuries in humans, wild and domestic animals^3,17^. Harmful species of trematodes forming host-parasite relationships with the radicines such as *Orientobilharzia turkestanica* (Skrjabin, 1913) and *Fasciola gigantica* Cobbold, 1855 may be mentioned here^18,19^. Despite their practical importance, the actual diversity of the radicine snails is still not described completely. The molecular studies undertaken in 2000–2010s have revealed a high level of cryptic diversity within this group^14,20,21^, but in most papers authored by molecular taxonomists only nameless clades or Molecular Operational Taxonomic Units (MOTUs) are presented, without any attempt to attach these molecular entities to nominal species previously described on the basis of the shell and soft body morphology. The overall species richness of the radicine group as well as the phylogenetic relationships within it are not known yet. The existing estimates of number of species of *Radix* and related taxa are disappointingly discordant, ranging from 5–6 to more than several dozens of species^5–8,17^.

### History of the taxonomy of the radicine pond snails

Three successive stages in the history of taxonomic studies on the radicine snails may be distinguished. The initial one was purely conchological, when all diagnoses of genera, subgenera, species, and infraspecific varieties were based exclusively on shells, their size, proportions, and coloration. Sometimes, such an approach had led researchers to accept a huge amount of varieties within a small number of species^22^ or even to elevate many of these varieties to the full species rank^23^.

The thorough studies of anatomical variation of *Radix* and other lymnaeids undertaken in the first half of the 20^th^ century^7,24,25^ revealed that the conchological variation in this family is enormous as compared to the anatomical one, and most species and varieties of radicine snails described during the 19^th^ century should be regarded as synonyms of a handful of valid taxa^26,27^. The qualitative differences in the soft body structure (especially those of the reproductive system) were laid as the basis of a new system, whereas the differences in shell features were regarded as insignificant for taxonomy and species identification^7,28^. Hubendick became the most influential taxonomist of this, second, historical stage of *Radix* systematics^7^. His taxonomic approach can be characterized as a hyper-lumping one. Hubendick accepted only two species of the radicine snails in the world fauna, *Lymnaea auricularia* and *L. peregra* (O.F. Müller, 1774), and he even refused to classify them as a subgenus of their own (Hubendick did not use the subgenus rank altogether)^7^. One of these taxa, *Lymnaea auricularia*, was treated by Hubendick as a ‘superspecies’, consisting of several geographical ‘races’^7^.

The morphological characters, both anatomical and conchological, are apparent and easy-to-obtain, however, their use is often biased due to subjective decisions and equivocal interpretations. For example, Kruglov and Starobogatov in Russia, who analyzed essentially the same characters of lymnaeids as Hubendick did (shells and reproductive morphology), could delineate several tens nominal species of radicines instead of two Hubendick’s species, and distributed them among as many as five subgenera (*Radix* s. str., *Peregriana* Servain, 1881, *Myxas*, *Cerasina*, and *Pacifimyxas* Kruglov & Starobogatov, 1985)^5,6^. The two former subgenera were further subdivided by Kruglov and Starobogatov into several ‘sections'^5,6^. This example demonstrates how desperately discrepant the morphological data may be.

The third, modern, stage of *Radix* taxonomy is characterized by predominance of molecular taxonomic^11,20,21,29–32^ and ‘integrative’^33–35^ taxonomic studies. It is commonly accepted today that the molecular data provide a reliable and helpful source of taxonomic and phylogenetic information that is able to correct taxonomic errors arising due to intra- and interspecific morphological variation and cases of parallel and convergent evolution. By means of molecular techniques, it was confirmed that the actual diversity of radicine snails is much higher than it was estimated by Hubendick^7^. For instance, von Oheimb *et al*.^21^ demonstrated that there is a remarkable cryptic diversity within *Radix* inhabiting the Tibetan Plateau, and several genetically defined clades, corresponding most probably to biological species (still undescribed) or even species complexes, occur there. In papers published by Aksenova *et al*.^33,34^ and Vinarski *et al*.^35^, the molecular support for some radicine species endemic to Northern Asia has been provided.

### Biogeographic and evolutionary studies of the radicine pond snails

Historically, most biogeographic and evolutionary studies of the radicines were morphology-based and focused on species inhabiting Europe that might have biased the conclusions. Recently, some works expanding the geographic coverage eastward, to the northern and central parts of Asia, have appeared^21,33–35^. In this work, we use the largest dataset ever analyzed that includes different radicine species sampled in various countries of Europe and Asia, without a bias towards studying the European populations.

Under the considerations mentioned above, this study aimed to clarify the phylogenetic and taxonomic structure of the Old World radicine lymnaeids by means of molecular genetic analysis of a large dataset including representatives of more than 30 biological species of this group, to define the modern genus-level clades, and to provide reliable information on their spatial distribution and biogeography. Taking into account the high levels of cryptic diversity in this group, our assessment of the species richness and distribution ranges is primarily based on the most comprehensive (both spatially and quantitatively) molecular dataset for the radicine pond snails sampled to date. Another important goal of our work was to designate the previously defined and still nameless molecular clades corresponding to either species or genera of the radicines by means of attaching available Latin names to these clades. We studied the type series and the original descriptions of many nominal species of *Radix* s. lato and related genera to find the correspondences between them and clades/MOTUs delineated in several recent molecular works^20,21,36^. A few radicine taxa are described here as new to science.

## Results

### Species richness of the radicine pond snails in the Old World based on the COI barcode data

**Figure 1:**
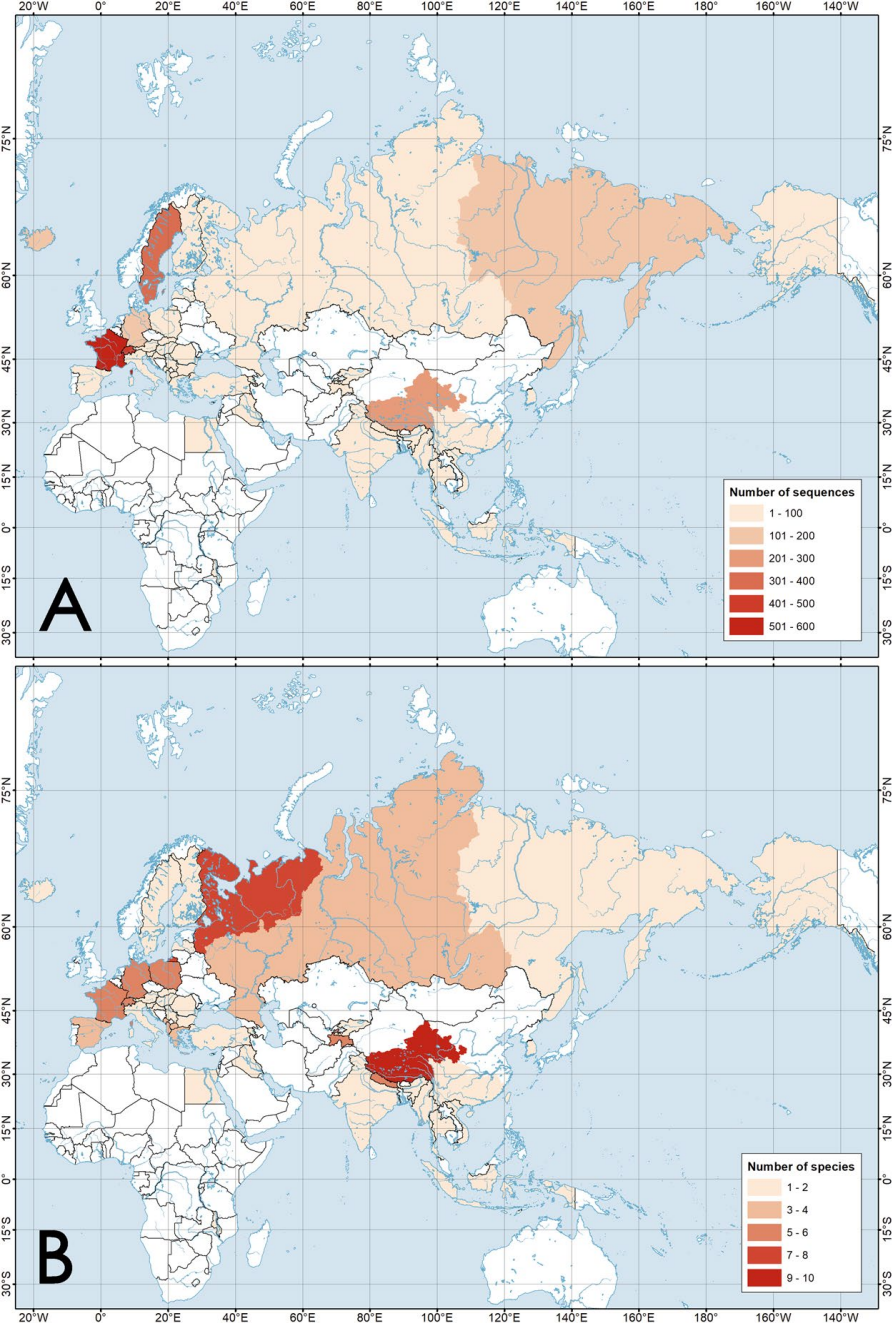
Spatial distribution of the number of available COI sequences and species richness of the radicine pond snails (Lymnaeidae: Amphipepleinae) across countries and regions of the Old World. (**A**) Map of the number of available COI sequences. (**B**) Map of species richness. The maps were created on the basis of Supplementary Dataset 1 using ESRI ArcGIS 10 software (www.esri.com/arcgis). The topographic base of the maps was created with Natural Earth Free Vector and Raster Map Data (www.naturalearthdata.com). (Maps: Mikhail Yu. Gofarov).

Our dataset comprises all the currently available COI sequences of the radicine pond snails (NCBI GenBank, BOLD IDS and our own data) and it represents the most comprehensive global sample of these molluscs analyzed to date (Fig. 1A,B, Supplementary Table 1 and Supplementary Dataset 1). This dataset contains 2,602 sequences from 38 countries, i.e. 34 Eurasian countries, 3 African countries and the USA (Supplementary Table 2 and Fig. 1A). There are no available sequences from 54 countries in Eurasia (58.7% of the Eurasian countries) and from 51 countries in Africa (94.4% of the African countries). The largest number of COI sequences is available from France, Switzerland, Sweden, China and Russia, although the two latter countries are actually not studied well, with sampling efforts mostly concentrated in a few areas (e.g. Tibet in China and the Far East in Russia). In summary, 77.4% of sequences in the dataset come from Europe (2,013 sequences) following by 21.2% from Asia (552 sequences), 0.9% from the Middle East (23 sequences), 0.5% from Africa (12 sequences) and 0.1% from North America (2 sequences).

The barcoding gap analysis reveals that there is a significant overlap between the distributions of intraspecific and interspecific distances (Supplementary Fig. 1). The multi-rate Poisson Tree Processes (mPTP) species-delimitation model calculated on the basis of the haplotype-level COI phylogeny (*N* = 1,382 unique haplotypes) returned 34 well-supported clusters corresponding to the putative species-level taxa (Supplementary Fig. 2). The majority of these clades were surely linked to certain nominal taxa on the basis of morphological and molecular evidences (Table 1 and Supplementary Note). However, available names seem to be lacking for six species inhabiting the high-altitude areas of the Tibetan Plateau. We were also unable to reveal available names for two species recorded from large lakes (Ohrid and Trichonis), but their taxonomic placement is in need of future investigations because they may represent local lacustrine populations of more widespread lineages.

**Table 1:** Taxonomic review of the radicine pond snails (Lymnaeidae: Amphipepleinae) in the Old World.

Genus	Species	Distribution range^*^
*Cerasina* Kobelt, 1881	*C. luteola* (Lamarck, 1822)	South Asia: India and Nepal
	*C. oxiana* (Boettger, 1889)	Central Asia: Tajikistan and South Asia: Nepal
	*C. siamensis* (Sowerby, 1873)	Southeast Asia: Myanmar and Thailand
*Radix* Montfort, 1810	*R. auricularia* (Linnaeus, 1758)	Widespread across mainland Eurasia (not recorded from Middle East, South Asia and Southeast Asia), Kurile Archipelago, Sakhalin, and Alaska
	*R. brevicauda* (Sowerby, 1873)	China: Western Tibet and Himalaya Range
	*Radix makhrovi* **sp**. **nov**.	China: Tibet, known from the Lake Donggi Cona system and the upstream of the Brahmaputra River basin west of the mouth of the Lhasa River, altitude range: 3,600-4,090 m
	*R. alticola* (Izzatullaev, Kruglov & Starobogatov, 1983)	Central Asia: Tajikistan and South Asia: Nepal
	*R. plicatula* (Benson, 1842)	Southeast Asia: Vietnam, South China: Yunnan, and Eastern Tibet: Gansu
	*R. euphratica* (Mousson, 1874)	Middle East: Iraq, Central Asia: Tajikistan, and Eastern Europe: European South of Russia
	*R*. sp. Trichonis	Greece: known from Lake Trichonis, but likely more widespread
	*R. rubiginosa* (Michelin, 1831)	Southeast Asia: Thailand, Singapore, and Indonesia up to Lesser Sundas (Flores), and Mascarenes: Réunion
	*R. natalensis* (Krauss, 1848)	Africa: Egypt, Malawi, and Cabo Verde Islands
	*R. rufescens* (Gray, 1822) = *R. acuminata* (Lamarck, 1822)	South Asia: India and Nepal, and Southeast Asia: Myanmar
*Ampullaceana* Servain, 1881	*A. ampla* (Hartmann, 1821)	Western Europe: Austria, Germany, Switzerland, and Eastern Europe: Montenegro and Poland
	*A. relicta* (Poliński, 1929) with two subspecies: *A. r. relicta* and *A. r. pinteri* (Schütt, 1974)**	Albania and Macedonia: endemic to Lakes Ohrid (*A. r. relicta*) and Prespa (*A. r. pinteri*)
	*A*. sp. Ohrid	Albania and Macedonia: known from southern feeder spring complexes of Lake Ohrid
	*A*. cf. *dipkunensis* (Gundrizer & Starobogatov, 1979)	Northern Europe: European North of Russia and Eastern Europe: Poland
	*A. lagotis* (Schrank, 1803)	Northern Europe: European North of Russia, Southern Europe: Greece (Crete), Central Asia: Tajikistan, and North Asia: Siberia
	*A. fontinalis* (Studer, 1820)	Western Europe: Germany, Switzerland, Eastern Europe: European South of Russia, Central and Volga regions of Russia, Bulgaria, Hungary, Poland, Northern Europe: European North of Russia, and Middle East: Turkey
	*A. balthica* (Linnaeus, 1758)	Widespread in Europe. Western Europe: Austria, France, Germany, United Kingdom, Spain, Switzerland, Northern Europe: Latvia, Sweden, Iceland, European North of Russia, and Eastern Europe: Poland. There is a single sequence from West Siberia
	*A. intermedia* (Lamarck, 1822)	Iberian Peninsula: France and Spain
*Peregriana* Servain, 1881	*P. dolgini* (Gundrizer & Starobogatov, 1979)	Widespread across Siberia, with a local population in the Pechora River basin in European North of Russia
	*P. peregra* (O.F. Müller, 1774) = *P. labiata* (Rossmässler, 1835)	Northern Europe: European North of Russia, Eastern Europe: Albania, Croatia, Macedonia, Montenegro, Serbia, Slovakia, Slovenia, Western Europe: France, Germany, Switzerland, Southern Europe: Greece, Italy, and Middle East: Turkey
*Kamtschaticana* Kruglov & Starobogatov, 1984	*K*. *kamtschatica* (Middendorff, 1851)	Widespread across North Asia: East Siberia and Russian Far East from Lake Baikal via the Amur River basin to Kamchatka
*Myxas* G. B. Sowerby I, 1822	*M*. *glutinosa* (O. F. Müller, 1774)	Northern Europe: Finland
*Tibetoradix* Bolotov, Vinarski & Aksenova **gen**. **nov**.	*T. hookeri* (Reeve, 1850) **gen**. **et comb**. **nov**.	China: Tibet, known from the upstream section of the Lhasa River and a single additional locality (Brahmaputra River basin), altitude range: 4,540-4,980 m
	*T. kozlovi* **sp**. **nov**.	China: Tibet, known from the Lake Donggi Cona system and the Requ Qu River (Yellow River basin), altitude range: 3,470-4,090 m
	*T*. sp.1	China: Tibet, known only from two sites, altitude range: 3,550-4,540 m
	*T*. sp.2	China: Tibet, known only from two sites, altitude range: 4,440-4,760 m
	*T*. sp.3	China: East Himalayan Mts., known only from two sites, altitude range: 3,920-4,460 m
	*T*. sp.4	China: Tibet, known only from a single lake, altitude: 4,310 m
*Orientogalba* Kruglov & Starobogatov, 1985	*O*. *ollula* (Gould, 1859)	East Asia: South Korea, South Asia: Nepal, and China: Tibet
	*O*. cf. *bowelli* (Preston, 1909)	China: Sichuan
	*O*. cf. *viridis* (Quoy & Gaimard, 1833)	Indonesia: Sumatra
Bullastra Bergh, 1901	*B. cumingiana* (L. Pfeiffer, 1855)^***^	Philippines

^*^Based on Supplementary Dataset 1. **Our model indicates that *Ampullaceana relicta* from Lake Ohrid and *A. pinteri* from Lake Prespa are conspecific due to the low level of genetic divergence (Supplementary Fig. 2). However, haplotypes of each taxon join into a subclade within the species-level clade that is in agreement with the model of Albrecht *et al*.^58^. With respect to their allopatric distribution ranges, morphological differences and putative ancient origin^58^, they are considered valid subspecies (Supplementary Note)^57^. ***Species has been included on the basis of our multi-locus phylogeny (Fig. 3).

We used a country-level approach to estimate the spatial patterns of species richness, but Russia and China were subdivided on several subregions because of their enormous areas (Supplementary Table 2). The mean species richness per country/subregion is relatively low (mean ± s.d. = 2.52 ± 1.92; *N* = 48) but this value is strongly biased by sampling effort (correlation between the number of species and number of available COI sequences per area: Spearman’s *r* = 0.664, *N* = 48, *p* < 0.001). The most species-rich areas are the Tibetan Plateau in China with 10 species following by Northern European Russia with 7 species (Fig. 1B and Supplementary Fig. 3). A total species richness in Eastern Europe is comparable with that in Tibet, with 10 species inhabiting each region (Supplementary Fig. 4). Africa and East Asia appear to be the most species-poor areas, with only one (*Radix natalensis*) and three (*Radix auricularia*, *Kamtschaticana kamtschatica* and *Orientogalba ollula*) species, respectively. However, our preliminary assessment reveals only most general diversity patterns of the radicine lymnaeids due to multiple gaps and poorly studied areas, including the well-known biodiversity hotspots such as Southeast China, North China, Pakistan, Afghanistan, Iran, Mongolia, Japan, Malaysia, Cambodia, Laos, Indonesia, the Philippines, and the entire African continent (Fig. 1A).

We revised previous molecular classification schemes of the radicine pond snails that were based on MOTUs^20^ and clades^21,36^ without links to certain biological species (Supplementary Table 3). A brief overview of all species of the radicines accepted here as valid, with taxonomical and nomenclatorial considerations as well as explanations of our taxonomic decisions can be found as Supplementary Note online. The full taxonomic account for these taxa, with illustrations of type specimens, measurements, diagnostic remarks and other information will be published elsewhere.

**Figure 2:**
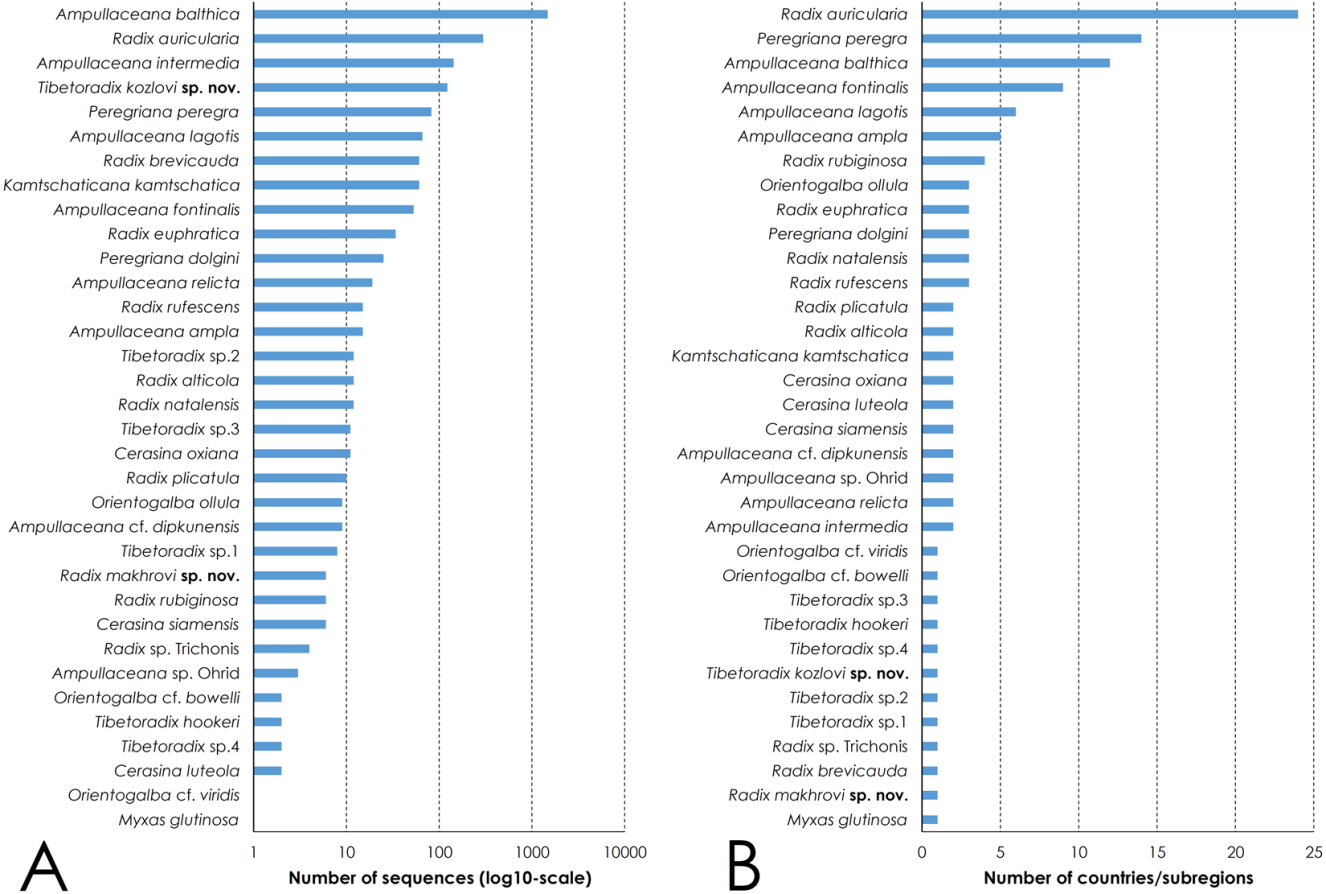
Species-level summary of the available COI sequences of the radicine pond snails (Lymnaeidae: Amphipepleinae) from 38 countries (Supplementary Dataset 1). (**A**) Number of available COI sequences per species. (**B**) Number of the country/subregion records of each species.

The number of available sequences per species in GenBank reflects rather the levels of sampling effort than the relative abundance of a species (Fig. 2A), with *Ampullaceana balthica*, *A. intermedia*, *Radix auricularia*, and *Tibetoradix kozlovi* **sp**. **nov**. being the most popular taxa (>100 sequences per species). *Radix auricularia*, *Ampullaceana balthica*, *A. fontinalis* and *A. lagotis* were recorded from the largest number of countries/subregions (Fig. 2B and Table 1). In contrast, 22 species were collected from the only one or two areas, e.g. *Radix makhrovi* **sp**. **nov**. and six *Tibetoradix* species are unknown outside the Tibetan Plateau.

### Major genus-level clades

**Figure 3:**
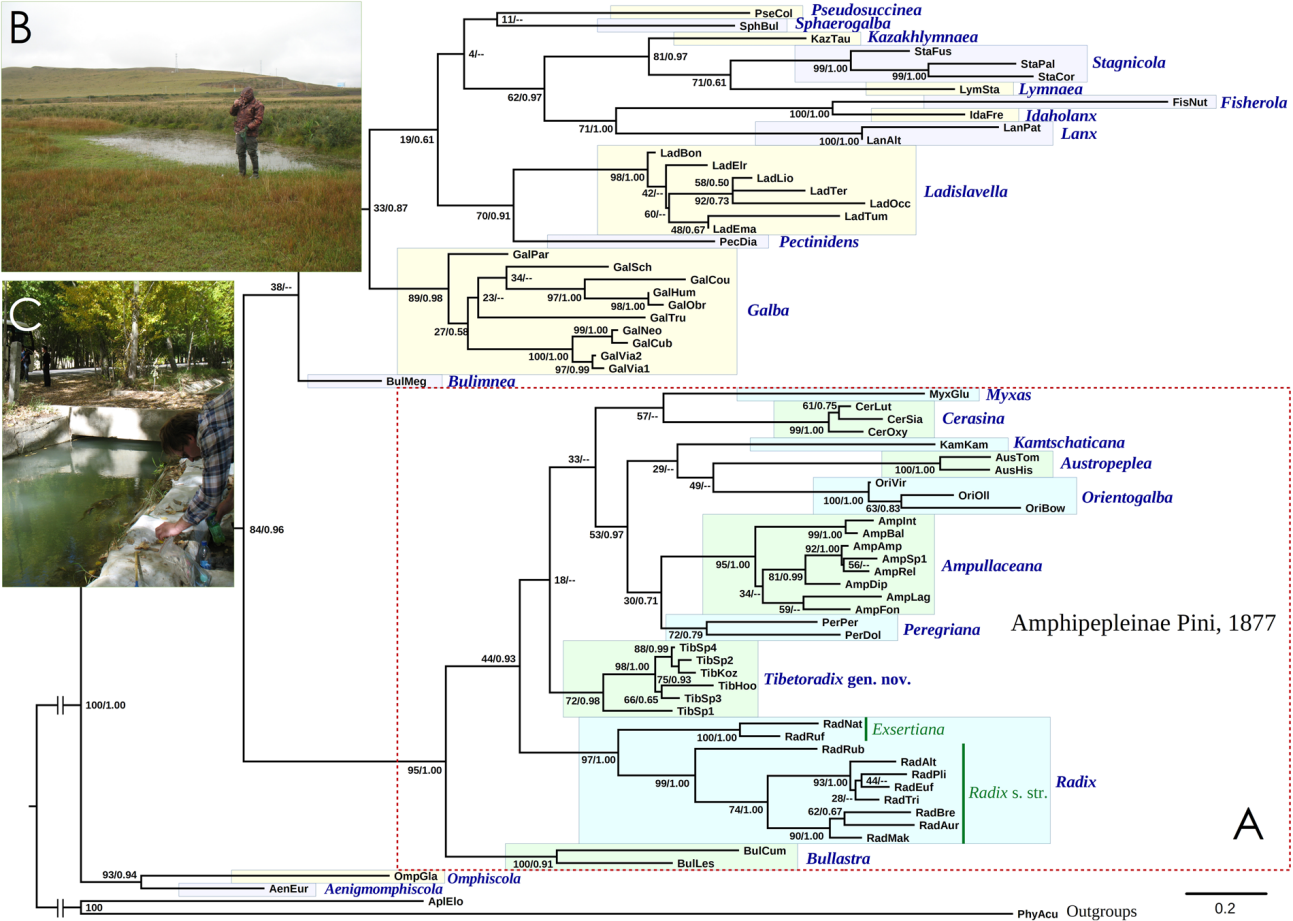
Global phylogeny of the radicine pond snails and the type localities of the two new species from the Tibetan Plateau. (**A**) Majority rule consensus phylogenetic tree of the Lymnaeidae recovered from RAxML analysis and obtained for the complete data set of mitochondrial and nuclear sequences (five partitions: three codons of COI + 16S rRNA + 28S rRNA). Black numbers near nodes are bootstrap support values/Bayesian posterior probabilities. The genus-level clades are highlighted in color. (**B**) Pool in the floodplain of the Requ Qu River (Yellow River basin), the type locality of *Tibetoradix kozlovi* **sp**. **nov**. (**C**) Human-made channel in the upstream of the Brahmaputra River west of the mouth of Lhasa, the type locality of *Radix makhrovi* **sp**. **nov**. (Photo: Valentina S. Artamonova).

**Figure 4:**
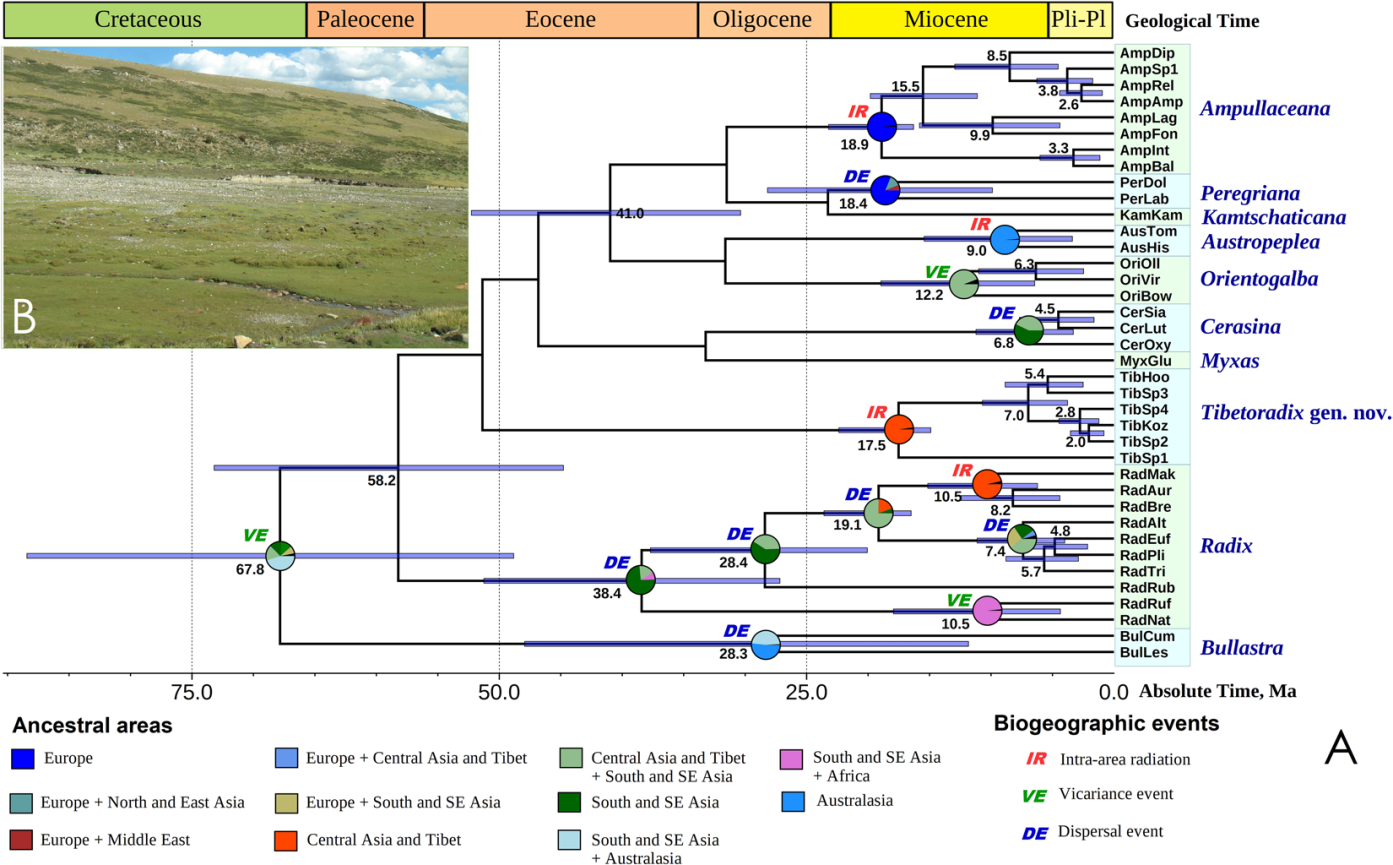
Divergence times and biogeography of the radicine pons snails. (**A**) Fossil-calibrated ultrametric chronogram of the Amphipepleinae calculated under a lognormal relaxed clock model and a Yule process speciation implemented in BEAST 1.8.4 and obtained for the complete data set of mitochondrial and nuclear sequences (five partitions: three codons of COI + 16S rRNA + 28S rRNA). The genus-level clades are highlighted in color. Bars indicate 95% confidence intervals of the estimated divergence times between lineages (Ma). Black numbers near nodes are mean ages (Ma). Stratigraphic chart according to the International Commission on Stratigraphy, 2015. The node pies indicate ancestral area reconstructions of the radicine lymnaeid clades (probability of each area combination) in accordance with the generalized biogeographic model (combination of the S-DIVA + DEC + S-DEC models). Age values and biogeographic reconstructions for weakly supported nodes (see Fig. 3) are omitted. (**B**) Upstream section of the Lhasa River Basin (Tibet), a habitat of *Tibetoradix hookeri* **gen**. **et comb**. **nov**. (Photo: Valentina S. Artamonova).

**Figure 5:**
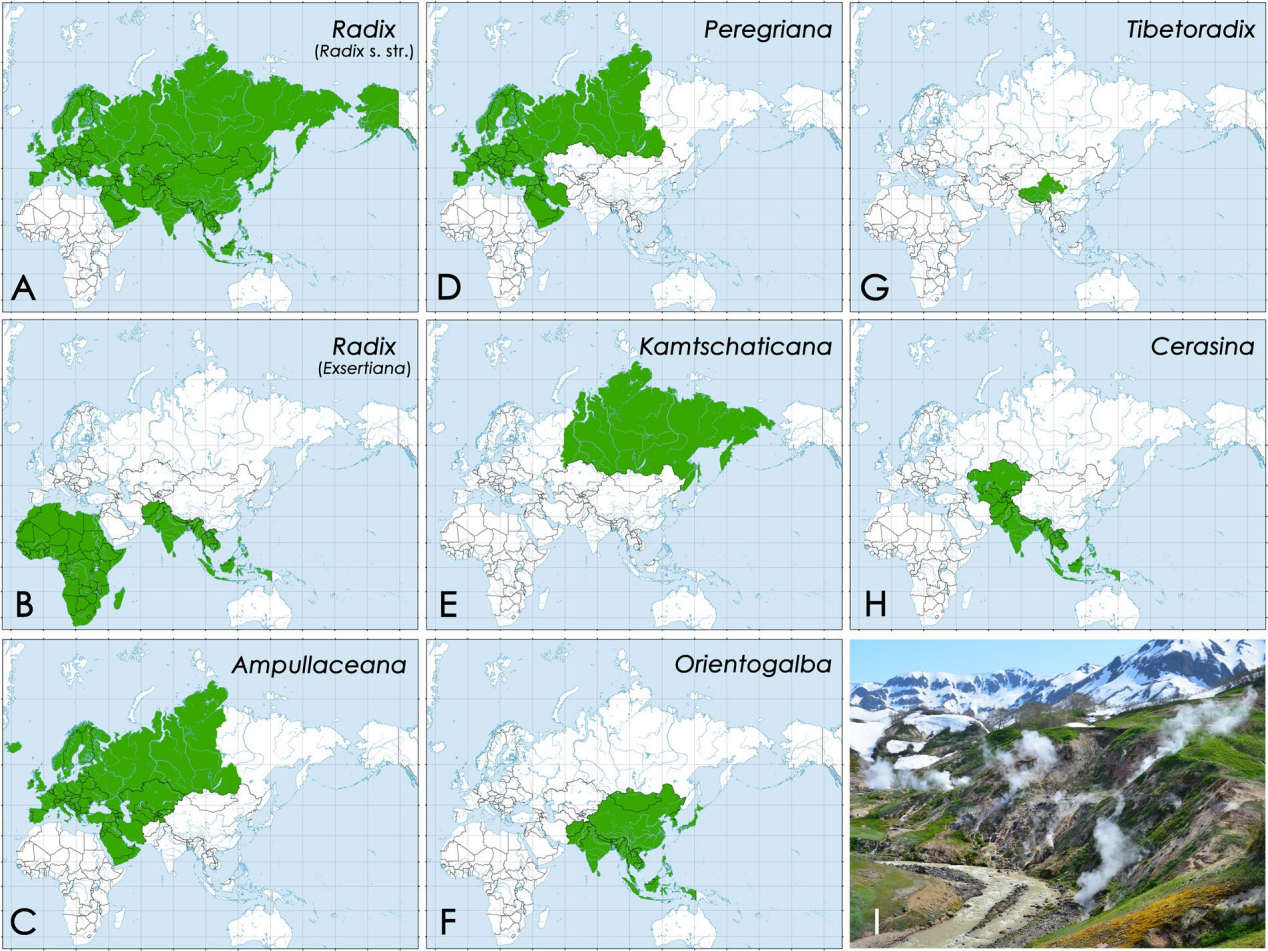
Maps of approximate geographic distribution of seven genera of the radicine pond snails in the Old World based on available COI sequences. The range of the genus *Myxas* is not shown due to a small amount of available COI sequences. The COI sequences of *Bullastra cumingiana* are not available, but this species is known only from the Philippines. (**A**,**B**) Subgenera of the *Radix*, i.e. (**A**) *Radix* s. str. and (**B**) *Exsertiana*. (**C**) *Ampullaceana*. (**D**) *Peregriana*. (**E**) *Kamtschaticana*. (**F**) *Orientogalba*. (**G**) *Tibetoradix* **gen**. **nov**. (**H**) *Cerasina*. The maps were created on the basis of Supplementary Dataset 1 using ESRI ArcGIS 10 software (www.esri.com/arcgis). The topographic base of the maps was created with Natural Earth Free Vector and Raster Map Data (www.naturalearthdata.com). (Maps: Mikhail Yu. Gofarov). (**I**) Habitat of *Kamtschaticana kamtschatica*: hot springs, warm puddles and geyser fields in the Valley of Geysers, Kamchatka. (Photo: Olga V. Aksenova).

Our multi-locus phylogeny reveals that the subfamily Amphipepleinae is a monophyletic entity (BS/BPP = 95/1.00) with ten well-supported and phylogenetically distant clades, the levels of divergence among which are comparable with those between genera of the Lymnaeinae (Figs 3 and 4). Almost all these clades are corresponding to available genus-level taxa, i.e. *Radix* Montfort, 1810, *Ampullaceana* Servain, 1881, *Peregriana* Servain, 1881, *Kamtschaticana* Kruglov & Starobogatov, 1984, *Orientogalba* Kruglov & Starobogatov, 1985, *Cerasina* Kobelt, 1881, and *Myxas* G. B. Sowerby I, 1822 from the Old World, *Bullastra* Bergh, 1901 from the Philippines and Australasia, and *Austropeplea* Cotton, 1942 endemic to Australasia. However, a single clade with six species inhabiting the Tibetan Plateau has no available name, and we therefore introduce a new genus, *Tibetoradix* Bolotov, Vinarski & Aksenova **gen**. **nov**. that is described here. The distribution range of each genus-level clade is presented in Taxonomic Account. The ranges of the Old World genera are illustrated in Fig. 5.

*Radix* is the largest genus with two subgenera, i.e., *Radix* s. str. and *Exsertiana* Bourguignat, 1883. The first subgenus contains three well-supported subclades representing the monophyletic species groups: (i) *Radix auricularia* group (*R. auricularia*, *R. brevicauda*, and *R. makhrovi* **sp**. **nov**.), (ii) *R. alticola* group (*R. alticola*, *R. plicatula*, *R. euphratica*, and *R*. sp. Trichonis), and (iii) the monotypic *R. rubuginosa* group (Supplementary Fig. 2). The interspecific relationships within the other large genus, *Ampullaceana*, are not so well resolved in our phylogeny, but, at the first glance, it could also be subdivided on three putative species groups: (i) *A. ampla* group (*A. ampla*, *A. relicta*, *A*. sp. Ohrid, and *A*. cf. *dipkunensis*, (ii) *A. balthica* group (*A. balthica* and *A. intermedia*), and (iii) *A. lagotis* group (*A. lagotis* and *A. fontinalis*).

The intergeneric relationships in our model are not resolved well, with the only *Radix* s. str. and *Exsertiana* surely being sister clades (BS/BPP = 97/1.00) (Fig. 3). Although our phylogeny indicates rather deep and complex evolutionary history of this large subfamily, in the absence of a robust reconstruction for deep nodes we are unable to establish a tribal-level taxonomic scheme for the Amphipepleinae, and it should be developed in a future, most likely on the basis of mitogenomic data.

### Statistical biogeography and divergence times of the radicine pond snails

Our fossil-calibrated phylogeny and generalized biogeographic model (Fig. 4 and Supplementary Table 4) indicate that the most recent common ancestor (MRCA) of the subfamily Amphipepleinae could have originated somewhere within a broad area covering Southeast Asia + Australasia near the Cretaceous/Paleocene boundary (probability = 45.3%; mean age = 67.8 Ma, 95% HPD 48.8–109.1 Ma). The MRCA of the *Radix* + *Exsertiana* clade most likely originated in Central Asia and Tibet in the Eocene (probability = 73.6%; mean age = 38.4 Ma, 95% HPD 27.2–51.2 Ma). The MRCA of *Radix* s. str. may have had a range in South and Southeast Asia but this scenario has a moderate support (probability = 59.3%). The crown group of this subgenus most likely evolved in the Oligocene (mean age = 28.4 Ma, 95% HPD 20.1–37.7 Ma). The *Radix auricularia* group most likely originated in Central Asia and Tibet in the Late Miocene (probability = 96.4%; mean age = 10.5 Ma, 95% HPD 6.2–15.1 Ma), whereas the place of origin of the *R. alticola* group remains uncertain (probability = 39.9% for a broad area covering Central Asia and Tibet + South and Southeast Asia). According to our fossil-calibrated model, the MRCA of the *Radix alticola* group could also have originated in the Late Miocene (mean age = 7.4 Ma, 95% HPD 4.0–11.1 Ma). The MRCA of the *Exsertiana* crown group may have had a broad distribution across South and Southeast Asia and Africa with next separation via a vicariance event in the Late Miocene (probability = 98.4%; mean age = 10.5 Ma, 95% HPD 4.4–17.9 Ma). The *Ampullaceana* and *Peregriana* clades originated in Europe with subsequent intra-area radiations (probability = 97.8% and 81.2%, respectively) starting in the Miocene (mean age = 18.9 Ma, 95% HPD 16.3–23.2 Ma and mean age = 18.4 Ma, 95% HPD 14.5–48.3 Ma, respectively). The crown group of *Tibetoradix* **gen**. **nov**. evolved on the Tibetan Plateau in the Miocene (probability = 97.9%; mean age = 17.5 Ma, 95% HPD 11.9–34.0 Ma). The MRCA of *Orientogalba* most likely had a broad ancestral range in Central and Southeast Asia in the Late Miocene (probability = 94.2%; mean age = 12.2 Ma, 95% HPD 9.9–28.2 Ma). The *Cerasina* MRCA may have evolved in South and Southeast Asia but with a moderate probability (probability = 57.2%), the crown group of this genus most likely originated in the Late Miocene (mean age = 6.8 Ma, 95% HPD 3.3–11.2 Ma). The *Bullastra* MRCA appears to be of an Australasian origin but this scenario has a moderate support (probability = 51.6%), the crown group of this genus could have originated in the Oligocene (mean age = 28.3 Ma, 95% HPD 11.8–47.9 Ma). Finally, the *Austropeplea* crown group could have originated in Australasia in the Late Miocene (probability = 94.2%; mean age = 9.0 Ma, 95% HPD 3.4–15.4 Ma).

**Taxonomic Account**

Family Lymnaeidae Rafinesque, 1815.

Type genus: *Lymnaea* Lamarck, 1799.

Subfamily Amphipepleinae Pini, 1877.

Type genus: *Amphipeplea* Nilsson, 1822 = *Myxas* G.W. Sowerby I, 1822.

**Genus** ***Cerasina*** **Kobelt, 1881.**

Type species: *Limnaea bulla* Kobelt, 1881 (by original designation).

**Figure 6:**
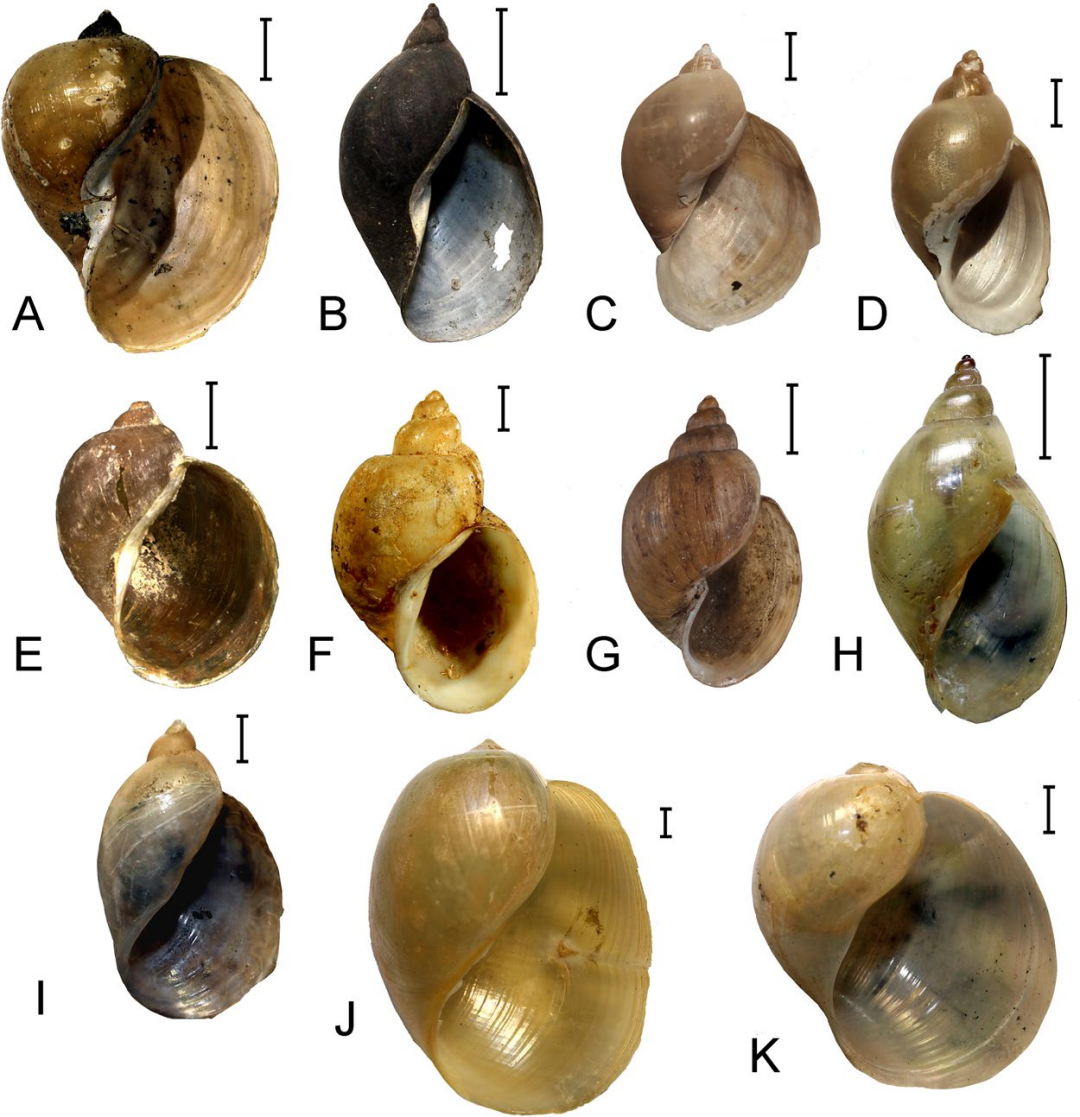
Examples of shell morphology of the radicine pond snails (Lymnaeidae: Amphipepleinae) from the Old World and Australasia. (**A**) *Radix* (*R*.) *auricularia*, Amur River basin, Russia (LMBI, M. Vinarski’s collection). (**B**) *Radix* (*Exsertiana*) *natalensis*, Undussuma, Congo (ZMB). (**C**) *Ampullaceana balthica*, Khar-Nuur Lake, Mongolia (LMBI, M. Vinarski’s collection). (**D**) *Peregriana peregra*, South Urals, Russia (ZISP). (**E**) *Kamtschaticana kamtschatica*, Kamchatka Peninsula, Russia (RMBH). (**F**) *Tibetoradix hookeri* **gen**. **et comb**. **nov**., a syntype, Tibet, China (NHMUK). (**G**) *Orientogalba viridis*, a syntype, Guam Island (NMNH). (**H**) *Cerasina oxiana*, Tajikistan (LMBI, M. Vinarski’s collection). (**I**) *Austropeplea tomentosa*, New Zealand (ZMB). (**J**) *Bullastra cumigiana*, Philippines (ZMB). (**K**) *Myxas glutinosa*, Lund, Sweden (ZMB, Westerlund’s collection). Scale bars: 2 mm (**C**–**G**,**I**,**J**,**K**) and 5 mm (**A**,**B**,**H**). (Photos: Maxim V. Vinarski [**A**–**D**,**F**–**K**] and Olga V. Aksenova [**E**]).

**Diagnosis**. Morphologically this genus is characterized by ovate-conical shell with smooth and glossy surface, relatively high spire and moderately inflated body whorl (Fig. 6H). The prostate with numerous (5–8) internal folds, a single synapomorphy to sharply distinguish *Cerasina* from all other genera of radicine snails (Supplementary Table 5). The spermathecal duct is rather long. The taxonomic identity of the type species of this genus is unclear that raises some nomenclatorial questions that will be discussed by us elsewhere.

**Distribution**. Three species of this genus are distributed in Central, South and Southeast Asia, from Turkmenistan and Tajikistan in the northwest to Thailand and Myanmar in the southeast (Fig. 5H, Table 1, and Supplementary Note).

**Genus** ***Myxas*** **G. B. Sowerby I, 1822.**

Type species: *Buccinum glutinosum* O. F. Müller, 1774 (by monotypy).

**Diagnosis**. The genus is characterized by globose, smooth and extremely fragile shell with very reduced spire (Fig. 6K), and the strongly developed mantle that in living snails covers the shell outside. The spermathecal duct is rather long, the prostate with a single fold inside.

**Distribution**. The range of the type species covers entire Europe (except of the southern parts) and extends into Western Siberia^37,38^. Some authors^6^ accept more species within *Myxas* but their validity is doubtful. Though we lack a broad set of sequences for *Myxas* (Table 1), the range of this genus may be defined well on the basis of morphologically-based recordings since phenotypically *Myxas* is so clearly distinct from all other lymnaeids of Europe and North Asia that it is almost impossible to confuse it with a species of another genus. The northeast Asian genus *Pacifimyxas* Kruglov & Starobogatov, 1985 remains enigmatical as its representatives have never been studied genetically, and both validity and phylogenetic affinities of *Pacifimyxas* are still not resolved.

**Genus** ***Radix*** **Montfort, 1810.**

Type species: *Radix auriculatus* Montfort, 1810 = *Helix auricularia* Linnaeus, 1758 (by original designation).

**Diagnosis**. Shell shape varies from auriculate to high conical, the body whorl and the aperture are usually well developed (Fig. 6A,B). The spire is usually short and acute. The prostate with a single internal fold, the spermathecal duct long. The subgenera *Radix* s. str. and *Exsertiana* Bourguignat, 1883 [type species is not designated; *Limnaeus exsertus* Martens, 1866 (=*Radix natalensis* auct.) is apparently the best candidate for the type species] are distant phylogenetic clades but both conchologically and anatomically indistinguishable from each other.

**Distribution**. This genus comprises not less than 10 valid species (Table 1 and Supplementary Note). It has the broadest native distribution range covering Eurasia, Africa, North America, the Indonesian Archipelago and the Indian Ocean Islands (Fig. 3B). This genus is known as far south as the Lesser Sundas and Mascarenes (*R. rubiginosa*) and South Africa (*R. natalensis*), and as far north as the Yamal Peninsula in Arctic Russia (*R. auricularia*). *R. auricularia* is the only representative of the subfamily in North America, with a possible native population in Alaska that seems to be the result of a recent (i.e. Holocene) dispersal event from the Russian Far East, whereas *R. natalensis* appears to be its single African member. However, the number of available sequences from Africa is very restricted, and records of several additional lineages in this continent could not be excluded. The subgenus *Radix* s. str. is widely distributed in Eurasia, with some species being geographically restricted to the Tibetan Plateau, including *Radix makhrovi* **sp**. **nov**. (Fig. 5A). The subgenus *Exsertiana* with two species, *R. rufescens* (Gray, 1822), and *R. natalensis* (Krauss, 1848), exhibits a disjunctive range (Fig. 5B). *R. natalensis* inhabits Africa and some adjacent countries such as the Arabian Peninsula, whereas *R. rufescens* is a tropical Asian lymnaeid distributed in India, Myanmar, Thailand and some other regions of Southeast Asia.

*Radix makhrovi* Bolotov, Vinarski & Aksenova **sp**. **nov**.

**Figure 7:**
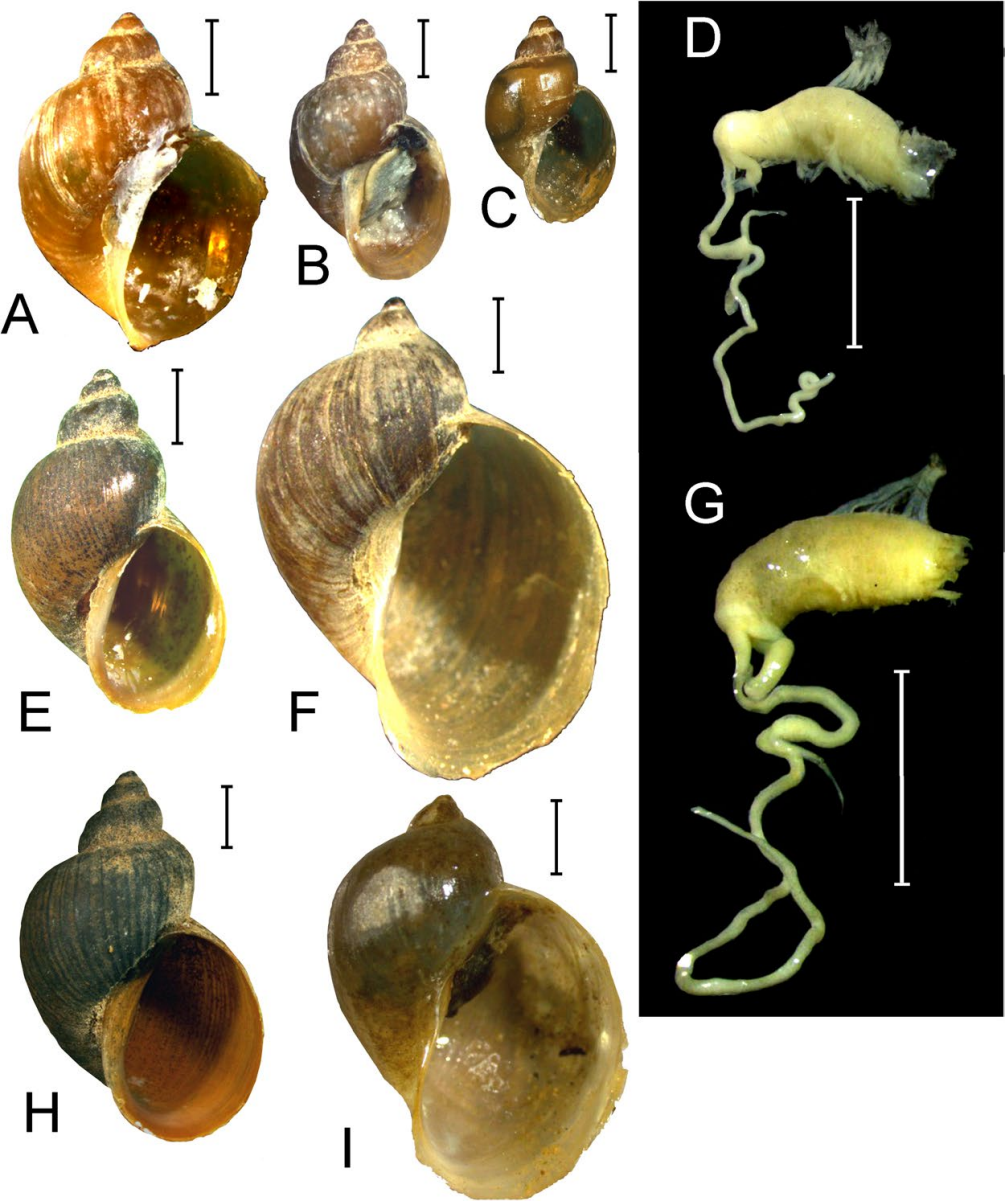
Shells and fragments of the genital system of lymnaeids endemic to Tibet. (**A**) *Tibetoradix kozlovi* **sp**. **nov**., the holotype, shell. (**B**,**C**) *T. kozlovi*, shells of two paratypes. (**D**) *T. kozlovi*, the copulatory apparatus of the holotype. (**E**,**H**) *T. hookeri* **gen**. **et comb**. **n**., Tibet, Lhasa River Basin, two shells. (**F**) *Radix makhrovi* **sp**. **nov**., the holotype. (**G**) *R. makhrovi* **sp**. **nov**., the copulatory apparatus of the holotype. (**I**) *R. makhrovi* **sp**. **nov**., a paratype. Scale bars 2 mm. (Photos: Olga V. Aksenova).

Figures 7F,G and I, Supplementary Tables 6 and 7.

**Type series**: The holotype and 2 paratypes are deposited in ZISP (accession numbers: 1–530/2018 and 2–530/2018, respectively), and 10 paratypes are deposited in RMBH (accession numbers: Mlym35 [5 specimens] and Mlym36 [5 specimens]). Holotype’s reference COI sequence no.: MH189861.

**Type locality**: China, Tibet, a roadside ditch west of the Lhasa River mouth, Brahmaputra River basin, 29°21′24″ N, 90°42′55″ E, alt. 3,597 m, 27.ix.2012, Makhrov & Artamonova leg.

**Etymology:** This taxon is dedicated to our colleague, Alexander A. Makhrov, a well-known Russian ichthyologist, who collected the type series of this species.

**Conchological diagnosis**: Shell brown, ovoid in outline, with small and relatively low spire and greatly expanded body whorl. Shell height is 11–16 mm, shell width constitutes 0.65–0.75 of shell height. Whorls slightly convex, separated by shallow suture, the tangential line visibly concave. The body whorl surface is covered by axial sculpture represented by more or less prominent growth lines. Aperture large, ovoid, with distinct angle in its upper part and evenly rounded basal margin. Columellar fold weakly developed, columellar lip narrow and thin, umbilicus closed. Shell measurements of the holotype (in mm): SH – 13.2; SW – 9.4; SpH – 3.4; BWH – 12.3; AH – 9.6; AW – 7.0. The holotype shell has 3.25 whorls. The measurements of the type series are presented in Supplementary Table 7.

**Reproductive anatomy**: The structure of the copulatory organ (see Fig. 7G) is typical for the radicines. Praeputium sac-like, oblong, light-colored, its width is almost the same along the entire length. The distal end of the praeputium is not inflated. The penis sheath is much narrower, its distal end is somewhat swollen. The lengths of the praeputium and the penis sheath are approximately equal. The spermathecal duct long. Prostate with a single fold inside.

**Differential diagnosis**: From the morphological point of view, i.e. both conchologically and anatomically, *R. makhrovi* **sp**. **nov**. exhibits no qualitative differences from some other species of this genus known from Tibet and Central Asia (for example, *Radix brevicauda*). Shell proportions of *R. makhrovi* as well as its spire and aperture shape are so characteristic for the subgenus *Radix* s. str. that we are inclined to regard this new taxon as a morphologically cryptic species which may be identified chiefly by means of molecular diagnosis (Supplementary Table 6).

**Distribution:** China: Tibet, known from the Lake Donggi Cona^36^ and the upstream of the Brahmaputra River west of the mouth of the Lhasa River.

**Genus** ***Ampullaceana*** **Servain, 1881.**

Type species: *Lymnaea ampullacea* Rossmässler, 1835 (? = *Radix balthica* auct.)

**Diagnosis**. The shell shape in snails of this group varies significantly, from almost globose or ear-shaped to ovate-conical; the spire of height and the relative size of the body whorl are also varied within the taxon (Fig. 6C). All snails in this genus are characterized by the prostate with a single internal fold and short spermathecal duct that sometimes may be virtually absent.

**Distribution**. This taxon embraces not less than 8 valid species, whose ranges encompass Europe, Iceland, Middle East, Siberia, and some parts of Central Asia (Table 1, Supplementary Note, and Fig. 5C).

**Genus** ***Peregriana*** **Servain, 1881.**

Type species: *Buccinum peregrum* O.F. Müller, 1774

**Diagnosis**. Conchologically and anatomically, this genus cannot be surely distinguished from the preceding taxon (Fig. 6D). Though the shell shape in *Peregriana* varies less widely than in *Ampullaceana*, and the absolute shell size in these two groups is different (*Peregriana* shells are a bit smaller than those of *Ampullaceana*), the extent of variation in shells and anatomical structures found in *Peregriana* greatly overlaps with variation in *Ampullaceana*. Therefore, this taxon may still be characterized by a molecular diagnosis only.

**Distribution**. We include two species into *Peregriana*: *P. peregra* and *P. dolgini* (Gundrizer & Starobogatov, 1979)^35^, the allopatric ranges of which cover Europe, Middle East and Siberia (Table 1, Supplementary Note, and Fig. 5D).

**Genus** ***Kamtschaticana*** **Kruglov & Starobogatov, 1984.**

Type species: *Lymnaea kamtschatica* Middendorff, 1851 (by original designation).

**Diagnosis**. A presumably monotypic genus (Table 1, Supplementary Note, and Fig. 6E); initially it was placed into *Peregriana* as one of ‘sections’ of the latter^39^. Later, it has been found that phylogenetically the two taxa are rather distant^34^, though both anatomically and conchologically *Kamtschaticana* may hardly be distinguished from *Peregriana*^5,6,17^.

**Distribution**. Geographically, *Kamtschaticana* is defined as a genus of northeast Asian distribution (Fig. 5E and Table 1), inhabiting the Kamchatka Peninsula (Fig. 5I), the Russian Far East, and adjacent parts of Eastern Siberia^34^.

**Genus** ***Tibetoradix*** **Bolotov, Vinarski & Aksenova gen. nov.**

Type species: *Lymnaea hookeri* Reeve, 1850 (type locality: “Thibetan or north side of Sikkim Himalaya, at 18,000 feet elevation”).

Figures 6F, 7A–E and H

**Etymology**. This genus is named after the greater Tibetan Plateau.

**Diagnosis**. Shell shape varies from ovoid to ovate-conical. Spire relatively large, prominent, the body whorl moderately inflated. Copulatory apparatus is of typical radicine structure. Prostate with a single fold inside. Spermatecal duct long. This genus represents a fairly distinct phylogenetic clade but there are no qualitative morphological traits to distinguish this genus from *Radix* s. str.

**Distribution**. This genus is endemic to the Tibetan Plateau (altitude range 3,550–4,980 m)^21,36^. It seems to comprise six species, most of which are known by a few specimens collected from one or two sites in Central Tibet (Table 1, Supplementary Note, and Fig. 5G). *Tibetoradix kozlovi* **sp**. **nov**. appears to be the most abundant and widespread species and it is described here. The other four possible undescribed taxa are in need of future research and additional sampling efforts.

*Tibetoradix kozlovi* Vinarski, Bolotov & Aksenova **sp**. **nov**.

Figure 7A–D, Supplementary Tables 6 and 7.

**Type series**: The holotype and 2 paratypes are deposited in ZISP (accession numbers: 1–531/2018 and 2–531/2018, respectively), and 21 paratypes are deposited in RMBH (accession number: MLym-685). Holotype’s reference COI sequence no.: MH190045.

**Type locality**: China, Central Tibet, the floodplain of the Requ Qu River, Yellow River basin, 33°35ʹ20.7ʹʹ N, 103°05ʹ30.2ʹʹ E, alt. 3,470 m, 15.ix.2017, Makhrov & Artamonova leg.

**Etymology**: This species is named in memory of Pyotr K. Kozlov (1863–1935), a famous Russian traveler and explorer of Central and East Asia (including Northern Tibet).

**Conchological diagnosis**: Shell small (SH up to 10 mm or a bit more), light brown-colored, translucent, ovoid to ovate-conical, fragile, with relatively high spire and well expanded body whorl. Spire conical, wide, its whorls are rounded and convex, sometimes almost shouldered, separated by a deep suture, the tangential line is virtually straight. Aperture relatively large, moderately expanded, ovoid, its basal margin is regularly rounded. Columellar lip wide and rather thin, it covers the umbilicus almost completely. Columellar fold weakly developed. Shell sculpture represented by growth lines of different prominence. Shell measurements of the holotype (in mm): SH = 10.6; SW = 7.6; SpH = 3.7; BWH = 9.1; AH = 6.8; AW = 5.0. The holotype shell has 4.0 whorls. The measurements of the type series are presented in Supplementary Table 7.

**Reproductive anatomy**: The structure of the copulatory organ (see Fig. 7D) is typical for the radicines. Praeputium cylindrical, oblong, light-colored, its width is almost the same along the entire length. The distal end of the praeputium is not inflated. The penis sheath is much narrower, its width grows slowly towards the distal end. The praeputium:penis sheath lengths ratio is roughly 1.50–1.75. The spermathecal duct long. Prostate with a single fold inside.

**Differential diagnosis**: Conchologically, this species resembles *T. hookeri* **gen**. **et comb**. **nov**. but may be distinguished from the latter by relatively lower spire and more expanded body whorl. Shell shape in *T. kozlovi* is ovoid (vs. ovate-conical), and shell is light brown, thin and translucent (vs. dark brown, thick and opaque) (see Fig. 7E,H). Besides, whorls of *T. hookeri* shells are more convex as compared to *T. kozlovi*. Juvenile individuals of *T. kozlovi* are almost indistinguishable from *T. hookeri* with respect to their shell proportions (compare Fig. 7B,E). This species differs from the other species in the genus by fixed nucleotide substitutions (Supplementary Table 6).

**Distribution**: The Great Tibetan Plateau. Perhaps, *T. kozlovi* is endemic to the Lake Donggi Cona^36^ and the upstream section of the Yellow River basin in Central Tibet.

**Genus** ***Orientogalba*** **Kruglov & Starobogatov, 1985**

=*Viridigalba* Kruglov & Starobogatov, 1985

Type species: *Lymnaea heptapotamica* Lazareva, 1967 (? = *Austropeplea ollula* Gould, 1859) (by original designation)

**Diagnosis**. From the anatomical viewpoint, *Orientogalba* is virtually identical to *Peregriana*, but it may be distinguished by much lesser size (up to 15 mm in height) and phenotypically it resembles the snails of the genus *Galba* belonging to another subfamily, Lymnaeinae (Fig. 6G). Shell shape varies from almost globose to turriculate, spermathecal duct long, prostate with a single fold inside.

**Distribution**. The genus includes at least 3 valid species (Table 1, Supplementary Note, and Fig. 5F) and it is widespread through the Pacific Region (Russian Far East to Australia), common in many countries of Central Asia (e.g., China and Mongolia), introduced into Spain^40^.

**Genus** ***Austropeplea*** **Cotton, 1942**

Type species: *Lymnaea aruntalis* Cotton & Godfrey, 1938 (by original designation)

**Diagnosis**. Shell shape varies from almost globose to ovoid, sometimes with rather high and distinct spire (Fig. 6I). Shell surface smooth, the mantle border is reflected^15,41^. Anatomically *Austropeplea* is very similar to *Radix*.

**Distribution**. The genus comprises several divergent species-level lineages from Australia and New Zealand^14^ but its actual species richness is still not known. In the Old World, this genus seems to be lacking.

**Genus** ***Bullastra*** **Bergh, 1901**

Type species: *Bullastra velutinoides* Bergh, 1901 (by monotypy).

**Diagnosis**. Shell fragile and globose, shell surface smooth, the spire is very low, sometimes greatly reduced (Fig. 6J). Similarly to *Austropeplea* and *Myxas*, the mantle border in these snails is reflected^41^. Anatomically, this genus is almost indistinguishable from *Radix*^15^.

**Distribution**. The range of this genus is south Pacific, with *B. cumingiana* (L. Pfeiffer, 1855) inhabiting the Philippines, and *B. lessoni* (Deshayes, 1831) occurring in Australia, New Zealand, and New Guinea^7,14^. The first species is a member of the Old World fauna.

## Discussion

### Molecular taxonomy and species richness of the radicine pond snails in the Old World

Identification of the radicine pond snails is a complicated task due to the high level of morphological variability and presence of numerous cryptic taxa^20,31–35,42^. A molecular approach appears to be the only way for reliable identification of species within this group^14,20^. However, the most popular molecular classification schemes of the radicine lymnaeids of the Old World were based on impersonal categories such as MOTUs^20^ and clades^21,34^, without links to the Latin binomial names demanded by the internationally adopted rules of zoological nomenclature. In the absence of an available phylogeny-based taxonomy, both the approaches were widely applying until recently^36,43,44^. To avoid the problems outlined above, we developed the first DNA barcode reference library of the Old World Amphipepleinae (Supplementary Dataset 1). This library contains 2,602 COI sequences of 34 biological species (all of the Old World species except for *Bullastra cumingiana*) and could be used as a key database for future taxonomic, phylogeographic, ecological and parasitological studies. Using this database, we linked all the MOTUs^20,43,44^ and clades^21,36^ to certain biological species and higher taxa (Supplementary Table 3). Finally, it partly allows a molecular identification of biological species using several other markers (e.g., 16S rRNA and ITS2) via specimens having the sequences of the COI and additional genes. The preliminary nation-level checklists of the radicine species in the Old World countries are presented in Supplementary Dataset 2. The lack of clear barcoding gap due to overlapping distributions of intraspecific and intraspecific distances indicates that a barcoding gap analysis likely represents an unsuitable tool for species delimitation in the radicine snails, as it was revealed for several other groups of invertebrates^45,46^.

Also we may report that there are much more species of the radicines than it was accepted in the most authoritative morphologically-based works of the past^7,8^, though the overall species richness is not so high as it was suggested by Kruglov & Starobogatov^5,6^. However, much work is needed to reach a more or less reliable estimate of the radicine species richness. Some taxa that may be included to this group on the basis of their conchological and anatomical characters (such as the genus *Pacifimyxas* Kruglov & Starobogatov, 1985 or *Lantzia* Jousseaume, 1872) have not been studied molecularly.

### Primary genus-level units of the radicine pond snails in the Old World

Our results indicate that the genus *Radix* s. lato should be split into a series of separate genera, each with own more or less distinct range, including some groups of rather limited distribution (e.g. *Tibetoradix* **gen**. **nov**.). The generic name *Radix* should be retained for a relatively compact group of species closely allied to *R. auricularia*. The scheme proposed by Vinarski^4^, with two subgenera within *Radix* (*Radix* s. str. and *Peregriana* Servain, 1881), is also too simple to reflect the actual diversity of the radicine snails at the above species level. Our generic classification is based on the divergent phylogenetic clades that were also recorded in many previous molecular works with broad taxon sampling but were not taxonomically defined^21,33–36^. Additionally, in our phylogeny *Bullastra* takes a very distant position from the other two Pacific genera (*Austropeplea* and *Orientogalba*) that is in agreement with the phylogenetic models of Puslednik *et al*.^14^.

### Evolutionary biogeography of the radicine pond snails

Our phylogenetic modelling suggests that the MRCA of the radicine clade could have originated near the Cretaceous – Paleocene boundary. Indeed there are reports of *Radix*-like shells from the Cretaceous deposits of China, Mongolia, and East Siberia^47^. Correa *et al*.^3^ suggested that the Indo-Pacific Region could be considered the old center of diversification of the radicine clade (*Austropeplea* + *Bullastra* + *Kutikina* + “*Radix*” + “*Lymnaea*”). This hypothesis is in agreement with our reconstructions that suggest rather a Southeast Asian – Australasian origin for this subfamily. The crown group of *Radix* appears to be the most ancient group, the origin of which is placed in the Eocene, while the MRCA of *Radix* s. str. and *Bullastra* most likely evolved in the Oligocene.

However, with respect to our models, the most intense radiation processes in different radicine lineages occurred in the Miocene. During this period, the crown groups of *Ampullaceana*, *Peregriana*, *Tibetoradix* **gen**. **nov**., *Orientogalba*, *Cerasina*, *Exsertiana*, as well as of the *Radix auricularia* and the *R. alticola* species groups were originated, and besides such a Miocene evolutionary explosion seems to be the primary cause shaping the modern diversity in the radicine pond snails. We suggest that warm and humid period during the Miocene Climatic Optimum (MCO) between 20 and 14 Ma^48^ have triggered the dispersal processes in freshwater snails, probably via passive transfer with water birds and active migrations through direct connections between freshwater basins. The extensive development of huge lake systems, e.g., in Europe and Central Asia, most likely supported successful range expansions in ancestral taxa during this period. Two great lake complexes covered as much as >150,000 km^2^ of the central Tibetan Plateau for several million years between 23.5 and 13.5 Ma^49^. During the Miocene humid intervals, vast freshwater systems were also presented in Central and South-Eastern Europe^50^, North Africa^51^, Australia^52^ and other regions.

The MCO ended abruptly between 14.0 and 13.5 Ma, with subsequent cool and dry climatic episode leading to major extinction events in thermophilic groups, e.g., amphibians^48^. Global climate cooling and Tibetan Plateau uplift caused enhanced aridification in Eurasia with disappearance of the Miocene lake complexes^49,53^. We suggest that the Late Cenozoic aridification could have induced radiation in the radicine lineages via fragmentation of their continuous ranges on multiple isolates in suitable refugia. This scenario is supported well by our biogeographic model indicating that the most important centers of origin and species-level diversification of the radicine pond snails were located in areas with the most extensive development of great Miocene lakes, i.e., Central Asia and Tibet (*Radix* s. str., *Tibetoradix* **gen**. **nov**. and probably *Orientogalba*) and Europe (*Ampullaceana* and *Peregriana*). Large lakes are well-known evolutionary hotspots, and paleontological studies revealed numerous, possible endemic fossil lineages of freshwater snails, including lymnaeids, from the Miocene lake systems^50^.

More recent divergence events in *Tibetoradix* **gen**. **nov**., *Cerasina* and *Ampullaceana* (Fig. 4) could have been caused by the transition from the warm Pliocene to the cold Pleistocene, strong climatic fluctuations during the Pleistocene^54^, and the appearance of several physical barriers between lineages, e.g., mountain ridges in Tibet^55^. Recent intense intraspecific radiations have been discovered in different lineages inhabiting specific environments, e.g., large lakes and geothermal springs^33,42,56–58^. Our results strongly support the hypothesis of the continuous existence of freshwater refugia for the radicine pond snails throughout the Last Glacial Maximum on the Tibetan Plateau^21,36^, but we suggest that suitable habitats have been existed there continuously from the Middle Miocene.

In summary, we propose a novel climate-dependent biogeographic model explaining the radicine radiations that predicts broad-scale dispersal events during the warm and humid episodes followed by the range fragmentation during the cold and dry periods. The ancient great lake complexes in Eurasia could have been served as primary radiation hotspots^50,59–64^ causing the Miocene evolutionary explosion in this group and shaping spatial patterns of recent species richness.

## Methods

### Data collection

To estimate the species richness and distribution of the radicine pond snails, we collected the dataset of 2,602 COI sequences of *Radix* spp. and related genera, including 191 new sequences and 2,411 sequences obtained from GenBank and BOLD IDS (Supplementary Dataset 1). From the public databases, 3,012 available COI sequences of the pond snails were collected using keywords “Lymnaeidae + COI” (Genbank) and “Lymnaeidae” (BOLD IDS) on 4 September 2017 but 600 sequences unrelated to the Amphipepleinae were excluded after preliminary molecular taxonomic analysis on the basis of maximum likelihood phylogeny reconstructed in MEGA6^65^. To construct the multi-locus phylogeny of the Lymnaeidae, we sampled the COI, 16S rRNA and 28S rRNA gene sequences for a single representative of each available species, including 24 new 28S rRNA sequences obtained in this study. The new sequences were generated and processed in accordance with the standard approach as described in our previous works^35,42,57^. The list of primer sequences^66–68^ is given in Supplementary Table 8.

### COI phylogeny and species delimitation

To estimate the putative species-level structure of our COI dataset, we aligned it using a MUSCLE algorithm^65^ and collapsed 2,602 sequences into 1382 unique haplotypes using an online FASTA sequence toolbox (FaBox 1.41)^69^. Haplotypes of *Omphiscola glabra* (O.F. Müller, 1774), *Pseudosuccinea columella* (Say, 1817) and *Lymnaea stagnalis* (Linnaeus, 1758) were used as an outgroup (Supplementary Dataset 1). This COI alignment was used to calculate a maximum likelihood phylogeny with RAxML v. 8.2.6 HPC Black Box^70^ at the San Diego Supercomputer Center through the CIPRES Science Gateway^71^ as described in Bolotov *et al*.^72^. The consensus COI phylogeny obtained from the RAxML analyses was used as an input tree for the mPTP species-delimitation model of Kapli *et al*.^73^ thorough online mPTP server (http://mptp.h-its.org). The novel mPTP approach appears to be the most appropriate and accurate method to delineate species boundaries within large phylogenies^73^. This model returns more general MOTUs than other available species-delimitation methods^72,73^ that is especially helpful to resolve species-level clades within the lymnaeids, because they may differ from other mollusc groups by elevated COI barcoding threshold and fast evolutionary rates^42,57^. Putative species-level clades were checked on the basis of morphological and biogeographic criteria and were compared with the type series and the original descriptions of nominal taxa to link each clade to a biological species (Supplementary Note).

### Multi-locus phylogeny and divergence time estimation

The datasets of the COI, 16S rRNA and 28S rRNA gene sequences containing a single haplotype per species were aligned using a MUSCLE algorithm^65^, were checked for the substitution saturation effects and conflicts of phylogenetic signals among gene partitions^46^, and were joined in the combined multi-gene nucleotide sequence alignment (Supplementary Table 9). Absent sites were treated as missing data. In phylogenetic analyses, we tested this three-locus alignment (five partitions: 3 codons of COI + 16S rRNA + 28S rRNA) with RAxML v. 8.2.6 HPC Black Box^70^ and MrBayes v. 3.2.6^74^ at the San Diego Supercomputer Center through the CIPRES Science Gateway^71^ as described in Bolotov *et al*.^72^. The best models of sequence evolution for each partition used in the Bayesian analyses are presented in Supplementary Table 10. For divergence time modelling, we developed a set of seven new calibrations, including six reliable fossil records^59–64,75,76^ with a supplement of one tectonic calibration^77^ (Supplementary Table 11). To compare the calibration lineages, we used the empirical scaling factor (ESF) of Marshall^78^ as described in Bolotov *et al*.^79^. An uncalibrated tree has been obtained as described in Vinarski *et al*.^80^, with two independent runs of BEAST v. 1.8.4, each with 50,000,000 generations. The ESF analysis revealed that all the calibrations are correspond well to the uncalibrated phylogenetic model (ESF > 45) (Supplementary Table 12). The time-calibrated phylogeny was calculated in BEAST v. 1.8.4^81^ with a lognormal relaxed clock algorithm, the Yule speciation process, and seven time calibrations (Supplementary Table 11) as the model priors. Calculations were performed at the San Diego Supercomputer Center through the CIPRES Science Gateway^71^. We specified the same evolutionary models as in the MrBayes analyses (Supplementary Table 10), but have used the simple HKY model instead of GTR^46,72^. Two replicate searches were conducted, each with 50,000,000 generations (sampling every 5,000 generation) and 10% burn-in, with subsequent processing as described in Bolotov *et al*.^46^ but with an additional resampling at every 10,000th generation. All the effective sample size (ESS) values were recorded as >1000.

### Ancestral area reconstruction

As the primary input data, we used a set of 9,000 three-locus ultrametric trees, which were obtained using BEAST v. 1.8.4 (see above). The ancestral area reconstruction model was calculated using three different approaches, i.e., Statistical Dispersal-Vicariance Analysis (S-DIVA), Dispersal-Extinction Cladogenesis (DEC, a Lagrange configurator), and Statistical Dispersal-Extinction Cladogenesis (S-DEC, a nonparametric ‘Bayes–Lagrange’ approach) implemented in RASP v. 3.2^82^ as described in Vinarski *et al*.^80^. Only the Amphipepleinae taxa were used for the modelling, with the distribution areas coded as follows: (a) Europe, (b) Middle East, (c) North and East Asia, (d) Central Asia and Tibet, (e) South and Southeast Asia, (f) Australasia, and (g) Africa. In addition to the models obtained from each analysis separately, we used the generalized model that combines the three other reconstructions.

### Studies of the type series of nominal taxa

The type specimens of various radicine species were studied in the malacological collections of the British Museum of Natural History, London, UK (NHMUK), National Museum of Natural History, Paris, France (NMNH), Zoological Institute of the Russian Academy of Sciences, Saint Petersburg, Russia (ZISP), and Natural History Museum of Berlin / Museum für Naturkunde, Germany (ZMB).

### Nomenclatural acts

The electronic edition of this article conforms to the requirements of the amended International Code of Zoological Nomenclature (ICZN), and hence the new names contained herein are available under that Code from the electronic edition of this article. This published work and the nomenclatural acts it contains have been registered in ZooBank (http://zoobank.org), the online registration system for the ICZN. The LSID for this publication is: urn:lsid:zoobank.org:pub:62383B9C-E0D3-4F4F-B833-AF405C039880. The electronic edition of this paper was published in a journal with an ISSN, and has been archived and is available from PubMed Central.

### Data availability

The sequences used in this study are available from GenBank. Accession numbers for each specimen are presented in Supplementary Dataset 1 and Supplementary Table 9. The type series of the two new species described here are available in ZISP – Zoological Institute of the Russian Academy of Sciences, Saint Petersburg, Russia and in RMBH – Russian Museum of Biodiversity Hotspots, Federal Center for Integrated Arctic Research of the Russian Academy of Sciences, Arkhangelsk, Russia.

## Electronic supplementary material

Supplementary Information

Supplementary Dataset 1

Supplementary Dataset 2
