# Supplementary material for: Species Richness, Molecular Taxonomy and Biogeography of the Radicine Pond Snails (Gastropoda: Lymnaeidae) in the Old World
Source: Sci Rep. 2018 Jul 25;8:11199. doi: 10.1038/s41598-018-29451-1 (PMC6060155; doi:10.1038/s41598-018-29451-1)
Supplement: Supplementary file 1 — Supplementary Information [file 41598_2018_29451_MOESM1_ESM.pdf]

SUPPLEMENTARY INFORMATION

Species Richness, Molecular Taxonomy and Biogeography of the Radicine Pond Snails (Gastropoda: Lymnaeidae) in the Old World

Olga V. Aksenova, Ivan N. Bolotov*, Mikhail Yu. Gofarov, Alexander V. Kondakov, Maxim V. Vinarski, Yulia V. Bespalaya, Yulia S. Kolosova, Dmitry M. Palatov, Svetlana E. Sokolova, Vitaly M. Spitsyn, Alena A. Tomilova, Oksana V. Travina, and Ilya V. Vikhrev

*Corresponding author: inepras@yandex.ru

Contents

Supplementary Figures

Supplementary Figure 1. Barcoding gap analysis of the radicine pond snails of the Old World and the USA based on the available *COI* sequences (Supplementary Dataset 1).

Supplementary Figure 2. Results of species delimitation analysis (mPTP model) based on the COI gene haplotype-level phylogeny of the Amphipepleinae. The list of haplotypes is given in Supplementary Dataset 1. The species-level clades supported by the modelling are collapsed. The *Ampullaceana balthica* clade is rolled up due to a huge amount of available haplotypes.

Supplementary Figure 3. Species richness of the radicine pond snails across countries/subregions of the Old World and the USA based on the available COI sequences (Supplementary Dataset 1).

Supplementary Figure 4. Species richness of the radicine pond snails across regions of the Old World and the USA based on the available COI sequences (Supplementary Dataset 1).

Supplementary Tables

Supplementary Table 1. Number of available COI sequences and generic and species richness of the radicine pond snails (Lymnaeidae: Amphipepleinae) in the Old World.

Supplementary Table 2. Number of available COI sequences and species richness of the radicine pond snails (Lymnaeidae: Amphipepleinae) across countries and regions of the Old World and the USA.

Supplementary Table 3. Taxonomic review of MOTUs and clades of the radicine pond snails (Lymnaeidae: Amphipepleinae) used in previous molecular works.

Supplementary Table 4. The most probable ancestral areas of the primary clades within the radicine pond snails inferred from three different statistical modeling approaches.

Supplementary Table 5. A comparison of conchological, anatomical, and caryological features of the genera and subgenera discussed in this paper.

Supplementary Table 6. Molecular diagnoses of two new radicine species from the Tibetan Plateau.

Supplementary Table 7. Morphometric characteristics (min-max / mean ± s.d.) of *Radix makhrovi* **sp. nov.** (type series), *Tibetoradix kozlovi* **sp. nov.** (type series), and *T. hookeri* **gen. et comb. nov.** (Tibet, Lhasa River Basin).

Supplementary Table 8. Primer sequences for PCR amplification and sequencing.

Supplementary Table 9. List of COI, 16S rRNA and 28S rRNA gene sequences used in phylogenetic reconstruction of the Lymnaeidae.

Supplementary Table 10. Models of sequence evolution for each partition based on corrected Akaike Information Criterion (AICc) of MEGA6 that were applied within a Bayesian inference framework.

Supplementary Table 11. List of fossil and tectonic calibrations that were used in BEAST analyses.

Supplementary Table 12. Evaluation of calibration lineages based on the empirical scaling factor (ESF).

Supplementary Notes

Supplementary Note. Systematic list of the Old World radicine taxa recovered during our study, with notes on their taxonomic position and nomenclature.

Supplementary Datasets

Supplementary Dataset 1. List of COI sequences used in this study, including species, haplotype, sampling locality and NCBI GenBank/BOLD IDS accession numbers [xlsx].

Supplementary Dataset 2. National checklists of the radicine pond snails (Lymnaeidae: Amphipepleinae) for countries and regions of the Old World based on the available COI sequences (1 January 2018) [pdf].

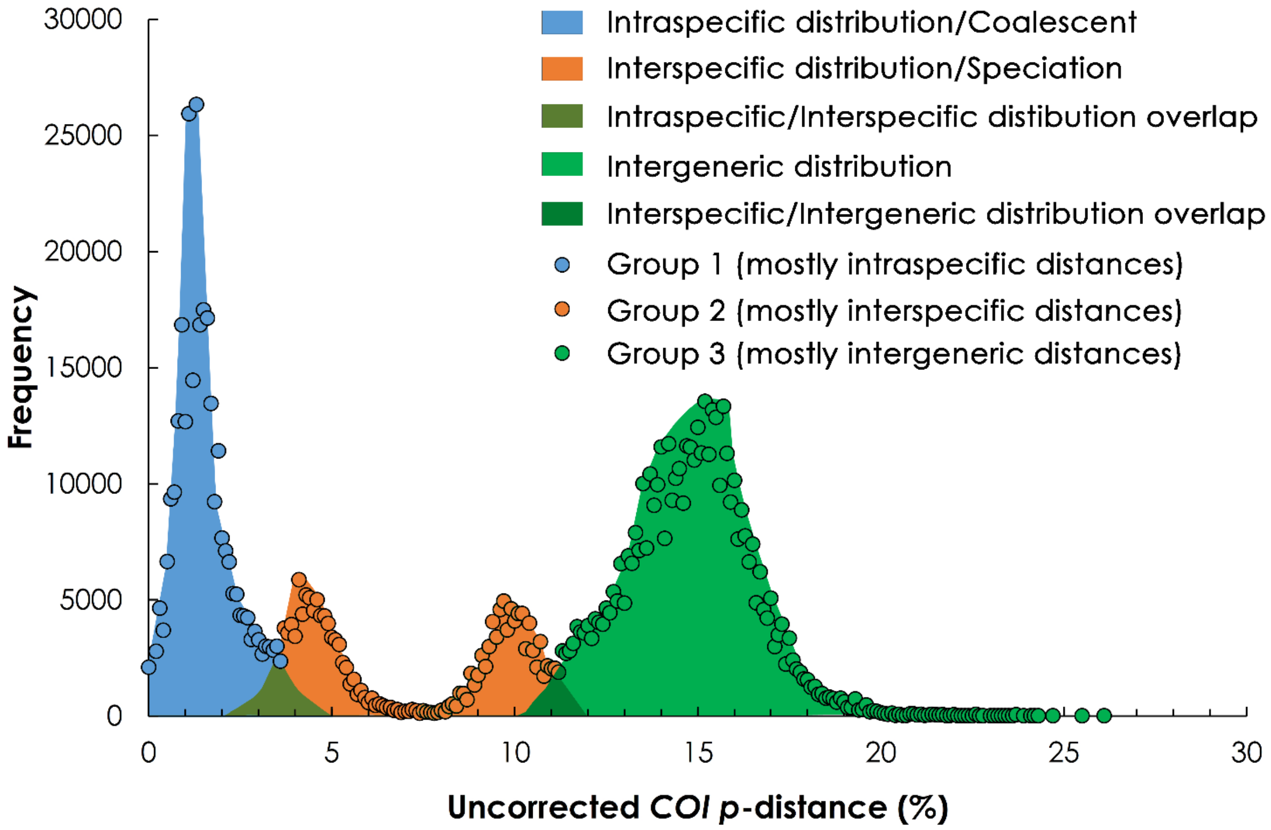

Supplementary Figure 1. Barcoding gap analysis of the radicine pond snails of the Old World and the USA based on the available *COI* sequences (Supplementary Dataset 1).

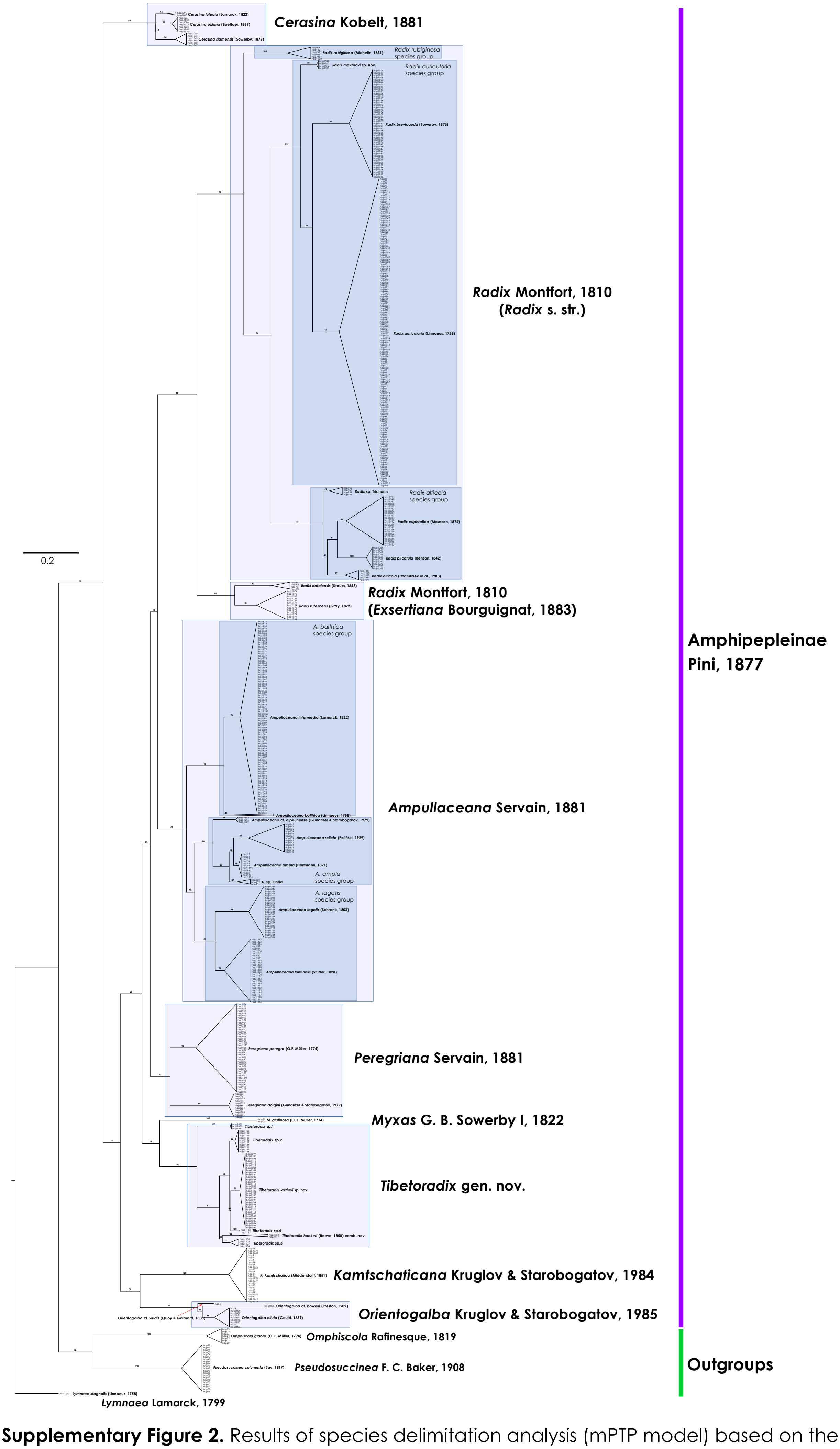

Supplementary Figure 2. Results of species delimitation analysis (mPTP model) based on the COI gene haplotype-level phylogeny of the Amphipepleinae. The list of haplotypes is given in Supplementary Dataset 1. The species-level clades supported by the modelling are collapsed. The *Ampullaceana balthica* clade is rolled up due to a huge amount of available haplotypes.

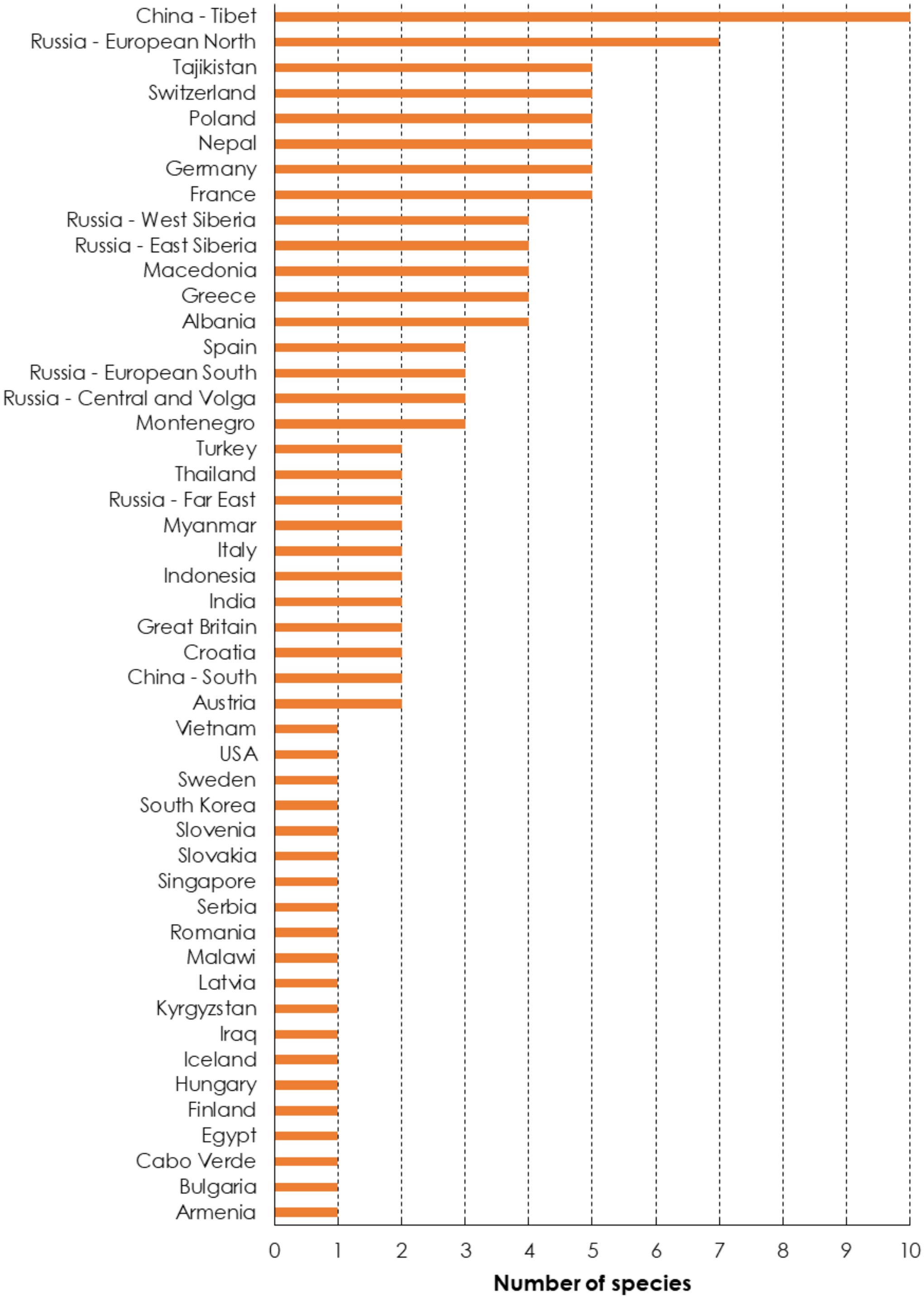

Supplementary Figure 3. Species richness of the radicine pond snails across countries/subregions of the Old World and the USA based on the available *COI* sequences (Supplementary Dataset 1).

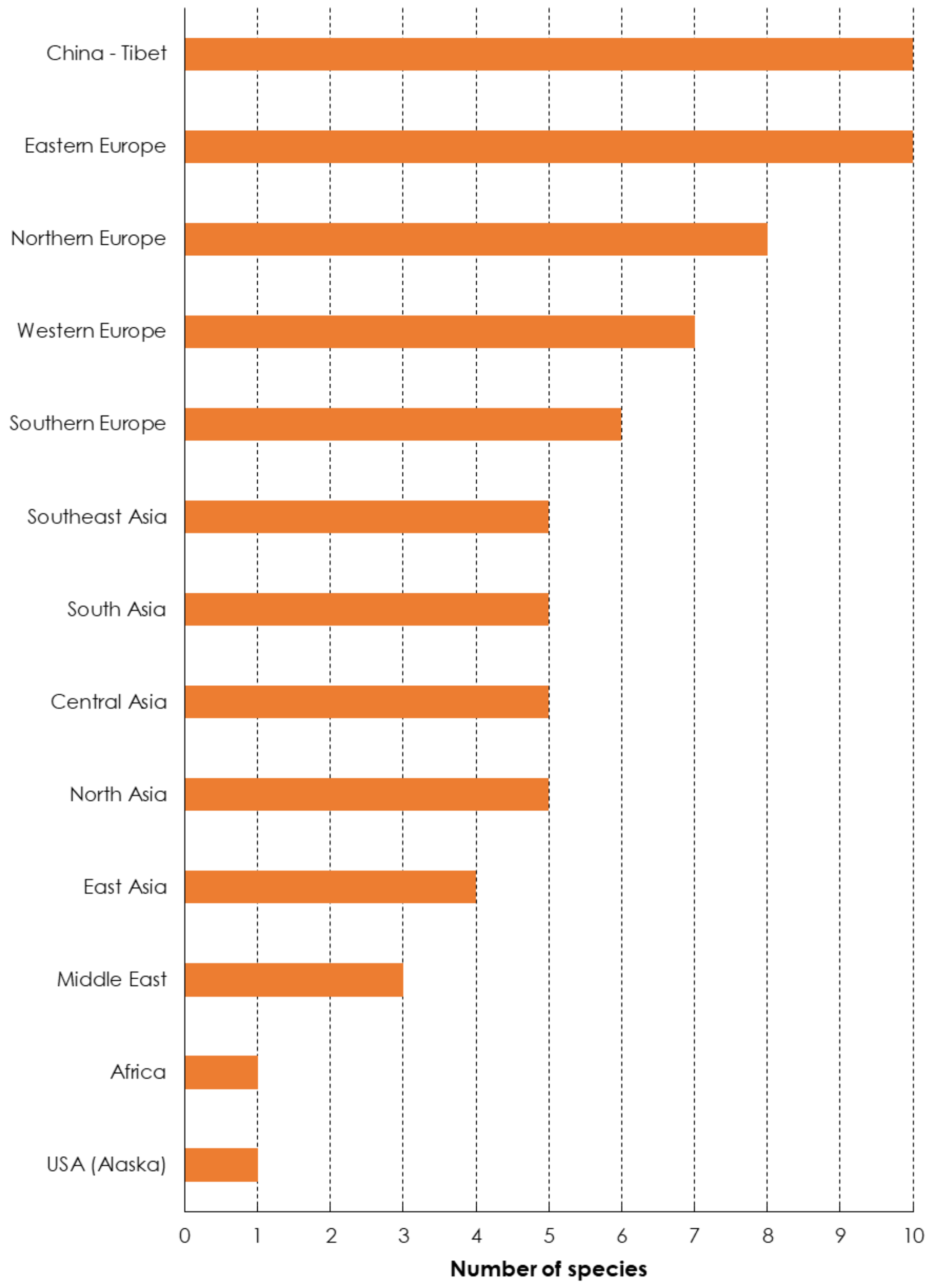

Supplementary Figure 4. Species richness of the radicine pond snails across regions of the Old World and the USA based on the available COI sequences (Supplementary Dataset 1).

Supplementary Table 1. Number of available COI sequences and generic and species richness of the radicine pond snails (Lymnaeidae: Amphipepleinae) in the Old World

Region	Number of sequences	Number of genera	Number of species
Northern Europe	487	4	8
Eastern Europe	110	3	10
Western Europe	1313	3	7
Southern Europe	103	3	6
Middle East	23	3	3
North Asia	173	4	5
Central Asia	44	3	5
Tibetan Plateau (China)	278	3	10
East Asia	15	2	4
South Asia	26	3	5
Southeast Asia	16	3	5
Alaska (USA)	2	1	1
Africa	12	1	1
**Total**	**2602**	**8**	**34**

Supplementary Table 2. Number of available COI sequences and species richness of the radicine pond snails (Lymnaeidae: Amphipepleinae) across countries and regions of the Old World and the USA. The list of sequences is given in Supplementary Dataset 1

Country	Region	Number of sequences	Number of species
Denmark	Northern Europe	0	0
Estonia	Northern Europe	0	0
Finland	Northern Europe	1	1
Iceland	Northern Europe	109	1
Latvia	Northern Europe	2	1
Lithuania	Northern Europe	0	0
Norway	Northern Europe	0	0
Russia - European North	Northern Europe	74	7
Sweden	Northern Europe	301	1
Albania	Eastern Europe	10	4
Armenia	Eastern Europe	4	1
Azerbaijan	Eastern Europe	0	0
Belarus	Eastern Europe	0	0
Bosnia and Herzegovina	Eastern Europe	0	0
Bulgaria	Eastern Europe	1	1
Croatia	Eastern Europe	3	2
Czech Republic	Eastern Europe	0	0
Georgia	Eastern Europe	0	0
Hungary	Eastern Europe	2	1
Kosovo	Eastern Europe	0	0
Macedonia	Eastern Europe	22	4
Moldova	Eastern Europe	0	0
Montenegro	Eastern Europe	3	3
Poland	Eastern Europe	40	5
Romania	Eastern Europe	2	1
Russia - European Central and Volga	Eastern Europe	8	3
Russia - European South	Eastern Europe	8	3
Serbia	Eastern Europe	1	1
Slovakia	Eastern Europe	6	1
Slovenia	Eastern Europe	1	1
Ukraine	Eastern Europe	0	0
Andorra	Western Europe	0	0
Austria	Western Europe	2	2
Belgium	Western Europe	0	0
France	Western Europe	542	5
Germany	Western Europe	193	5
Ireland	Western Europe	0	0

Country	Region	Number of sequences	Number of species
Liechtenstein	Western Europe	0	0
Luxembourg	Western Europe	0	0
Monaco	Western Europe	0	0
Netherlands	Western Europe	0	0
Switzerland	Western Europe	463	5
United Kingdom	Western Europe	113	2
Cyprus	Southern Europe	0	0
Greece	Southern Europe	8	4
Italy	Southern Europe	3	2
Malta	Southern Europe	0	0
Portugal	Southern Europe	0	0
San Marino	Southern Europe	0	0
Spain	Southern Europe	91	3
Bahrain	Middle East	0	0
Iran	Middle East	0	0
Iraq	Middle East	20	1
Israel	Middle East	0	0
Jordan	Middle East	0	0
Kuwait	Middle East	0	0
Lebanon	Middle East	0	0
Oman	Middle East	0	0
Palestine	Middle East	0	0
Qatar	Middle East	0	0
Saudi Arabia	Middle East	0	0
Syria	Middle East	0	0
Turkey	Middle East	3	2
United Arab Emirates	Middle East	0	0
Yemen	Middle East	0	0
Russia - West Siberia and Urals	North Asia	12	4
Russia - East Siberia	North Asia	61	4
Russia - Far East	North Asia	101	2
Kazakhstan	Central Asia	0	0
Kyrgyzstan	Central Asia	2	1
Tajikistan	Central Asia	42	5
Turkmenistan	Central Asia	0	0
Uzbekistan	Central Asia	0	0
China - Tibet	Tibet & Qinghai	278	10
China - South [Sichuan, Yunnan and regions east to]	East Asia	11	2
China - North and Northeast	East Asia	0	0
Japan	East Asia	0	0

Country	Region	Number of sequences	Number of species
Mongolia	East Asia	0	0
North Korea	East Asia	0	0
South Korea	East Asia	3	1
Taiwan	East Asia	0	0
Afghanistan	South Asia	0	0
Bhutan	South Asia	0	0
India	South Asia	2	2
Maldives	South Asia	0	0
Nepal	South Asia	24	5
Pakistan	South Asia	0	0
Sri Lanka	South Asia	0	0
Bangladesh	Southeast Asia	0	0
Brunei	Southeast Asia	0	0
Cambodia	Southeast Asia	0	0
Indonesia	Southeast Asia	3	2
Laos	Southeast Asia	0	0
Malaysia	Southeast Asia	0	0
Myanmar	Southeast Asia	8	2
Philippines	Southeast Asia	0	0
Singapore	Southeast Asia	1	1
Thailand	Southeast Asia	3	2
Vietnam	Southeast Asia	1	1
Malawi	Africa	1	1
Egypt	Africa	10	1
Cabo Verde	Africa	1	1
USA (Alaska)	North America	2	1

Supplementary Table 3. Taxonomic review of MOTUs[1] and clades[2,3] of the radicine pond snails (Lymnaeidae: Amphipepleinae) used in previous molecular works

**Genus**	**Species**	**MOTUs of Pfenninger et al.**[1]	**Clades of von Oheimb et al.**[2] **and Clewing et al.**[3]
Cerasina Kobelt, 1881	*C. luteola* (Lamarck, 1822)	N/A	Clade 11
	C. oxiana (Boettger, 1889)	N/A	Clade 11
	C. siamensis (Sowerby, 1873)	N/A	Clade 11
Radix Montfort, 1810	*R. auricularia* (Linnaeus, 1758)	MOTU4	Clade 1
	R. brevicauda (Sowerby, 1873)	N/A	Clade 2
	R. makhrovi **sp. nov.**	N/A	Clade 13
	R. alticola (Izzatullaev, Kruglov & Starobogatov, 1983)	N/A	Clade 3
	R. plicatula (Benson, 1842)	N/A	Clade 3
	R. euphratica (Mousson, 1874)	N/A	Clade 3
	R. sp. Trichonis	N/A	Clade 3
	R. rubiginosa (Michelin, 1831)	N/A	N/A
	R. natalensis (Krauss, 1848)	N/A	Clade 4
	R. rufescens (Gray, 1822)<br>=*R. acuminata* (Lamarck, 1822)	N/A	Clade 5
Ampullaceana Servain, 1881	*A. ampla* (Hartmann, 1821)	Clade 6: BIA2	Clade 8
	A. balthica (Linnaeus, 1758)	MOTU2	N/A
	A. intermedia (Lamarck, 1822)	MOTU3	N/A
	A. lagotis (Schrank, 1803)	N/A	Clade 7
	A. relicta (Poliński, 1929)	Clade 6: *Radix relicta* + *R. pinteri*	Clade 8
	A. fontinalis (Studer, 1820)	MOTU1	Clade 7
	A. sp. Ohrid	N/A	Clade 8
	A. cf. *dipkunensis* (Gundrizer & Starobogatov, 1979)	Clade 6: JEG2	N/A
Peregriana Servain, 1881	*P. dolgini* (Gundrizer & Starobogatov, 1979)	N/A	N/A
	P. peregra (O.F. Müller, 1774)<br>=*P. labiata* (Rossmässler, 1835)	MOTU5	Clade 6
Kamtschaticana Kruglov & Starobogatov, 1984	*K. kamtschatica* (Middendorff, 1851)	N/A	N/A
Myxas G. B. Sowerby I, 1822	*M. glutinosa* (O. F. Müller, 1774)	N/A	N/A
Tibetoradix Bolotov, Vinarski & Aksenova **gen. nov.**	*T. hookeri* (Reeve, 1850) **gen. et comb. nov.**	N/A	Clade 9
	T. kozlovi **sp. nov.**	N/A	Clade 9
	T. sp.1	N/A	Clade 10
	T. sp.2	N/A	Clade 9
	T. sp.3	N/A	Clade 9
	T. sp.4	N/A	Clade 9

Genus	Species	MOTUs of Pfenninger et al.[1]	Clades of von Oheimb et al.[2] and Clewing et al.[3]
Orientogalba Kruglov & Starobogatov, 1985	*O. ollula* (Gould, 1859)	N/A	Clade 12
	O. cf. *bowelli* (Preston, 1909)	N/A	Clade 12
	O. cf. *viridis* (Quoy & Gaimard, 1833)	N/A	N/A

N/A – species was lacking in the corresponding COI dataset.

Supplementary Table 4. The most probable ancestral areas of the primary clades within the radicine pond snails inferred from three different statistical modeling approaches

Clades	Ancestral areas	Type of biogeographic events	Probability of ancestral areas (%)*			
			S-DIVA	DEC	S-DEC	Generalized model
Amphipepleinae	South and Southeast Asia + Australasia	Vicariance	**72.4**	35.4	28.2	45.3
Cerasina	South and Southeast Asia	Dispersal	**93.0**	67.9	46.4	57.2
Radix	Central Asia and Tibet	Dispersal	**99.7**	56.2	64.9	**73.6**
Radix (*Radix* s. str.)	South and Southeast Asia	Dispersal	57.2	56.8	63.9	59.3
Radix auricularia group + *Radix alticola* group	Central Asia and Tibet + South and Southeast Asia	Dispersal	57.2	**88.0**	**80.2**	**75.1**
Radix auricularia group	Central Asia and Tibet	Intra-area radiation	**99.5**	**100.0**	**89.8**	**96.4**
Radix alticola group	Central Asia and Tibet + South and Southeast Asia	Dispersal	34.8**	69.7	40.9	39.9
Radix (*Exsertiana*)	South and Southeast Asia + Africa	Vicariance	**100.0**	**100.0**	**95.3**	**98.4**
Ampullaceana	Europe	Intra-area radiation	**100.0**	**100.0**	**93.4**	**97.8**
Peregriana	Europe	Intra-area radiation	**100.0**	**70.9**	**72.7**	**81.2**
Orientogalba	Central Asia and Tibet + South and Southeast Asia	Vicariance	**95.5**	**100.0**	**87.1**	**94.2**
Tibetoradix **gen. nov.**	Tibet	Intra-area radiation	**100.0**	**100.0**	**93.7**	**97.9**
Bullastra	Australasia	Dispersal	**88.5**	66.4	65.1	51.6
Austropeplea	Australasia	Intra-area radiation	**100.0**	**100.0**	**96.7**	**98.9**

*High support values (probability ≥70 %) are highlighted in bold. **South and Southeast Asia.

Supplementary Table 5. A comparison of conchological, anatomical, and caryological features of the genera and subgenera discussed in this paper

Genus (Subgenus)	Shell height	Shell shape	Shell surface	Spire	Spermathecal duct	Mantle border	Haploid chromosome number	Prostate
Cerasina	Up to 25 mm	Ovoid to ovate-conical	Smooth	High and relatively wide	Long	?Reflected	?17	Multifolded
Myxas	Up to 25 mm	Globose	Smooth	Flattened, very low	long	Reflected	17	With a single fold
Radix s. str.	Up to 35 mm	Ear-shaped to ovate-conical	Sculptured	Low and acute	Long	Not reflected	17	With a single fold
Exsertiana	Up to 30 mm	Ovate-conical	Sculptured	Low and acute	Long	Not reflected	17	With a single fold
Ampullaceana	Up to 25 mm	Ear-shaped to conical	Sculptured	Low to relatively high and obtuse	Short	Not reflected	17	With a single fold
Peregriana	Up to 25 mm	Ovate-conical to conical	Sculptured	Medium-sized and obtuse	Short	Not reflected	17	With a single fold
Kamtschaticana	Up to 20 mm	Ovate-conical to almost globose	Sculptured	Low and obtuse	Short	Not reflected	?17	With a single fold
Tibetoradix **gen. nov.**	Up to 15 mm	Ovate-conical	Sculptured	High to medium-sized, narrow and obtuse	Long	Not reflected	?17	With a single fold
Orientogalba	Up to 15 mm	Ovate-conical to almost globose	Sculptured	High to low, obtuse	Long	Not reflected	16	With a single fold
Austropeplea	Up to 30 mm	Globose to ovoid	Smooth	Low to moderately raised, obtuse	Long	Reflected	16	With a single fold
Bullastra	Up to 30 mm	Globose	Smooth	Very low, reduced. obtuse	long	Reflected	16	With a single fold

*The data were taken from the following sources: Hubendick[4], Correa et al.[5], Patterson and Burch[6], Kruglov and Starobogatov[7-9], Ponder and Waterhouse[10], Kruglov[11], and Vinarski[12]. Our own observations on some taxa (*Cerasina*, *Orientogalba*, and *Tibetoradix* **gen. nov.**) were also used.

Supplementary Table 6. Molecular diagnoses of two new radicine species from the Tibetan Plateau

Species	Nearest neighbors (mean COI p-distance ± standard error estimate, %)	Fixed nucleotide differences*	
		COI	16S rRNA
Tibetoradix kozlovi **sp. nov.**	*T.* sp.2 (2.1±0.6), *T.* sp.4 (2.6±0.7), *T.* sp.3 (4.3±0.7), *T. hookeri* **gen. et comb. nov.** (6.2±0.9), *T.* sp.1 (8.3±1.1)	50G, 521A, 546C	42A, 43C, 190G, 193C
Radix makhrovi **sp. nov.**	*R. auricularia* (8.7±1.0), *R. brevicauda* (9.0±1.1)	29G, 32C, 60T, 233C, 347A, 401T, 416G, 476G	N/A

*Based on the sequence alignment of the *Tibetoradix* spp. (for *T. kozlovi* **sp. nov.**) and the *Radix auricularia* species group (for *R. makhrovi* **sp. nov.**). N/A – not available.

Supplementary Table 7. Morphometric characteristics (min-max / mean ± s.d.) of *Radix makhrovi* **sp. nov.** (type series*), *Tibetoradix kozlovi* **sp. nov.** (type series*), and *T. hookeri* **gen. et comb. nov.** (Tibet, Lhasa River Basin)

**Measurement/Index**	**Species**		
	***R. makhrovi* sp. nov. (*N* = 10)**	***T. kozlovi* sp. nov. (*N* = 11)**	***T. hookeri* gen. et com. nov. (*N* = 7)**
Shell height (SH), mm	11.4–15.9<br>12.7±1.2	7.6–10.6<br>9.2±1.3	9.1–14.3<br>11.9±1.6
Shell width (SW), mm	7.9–10.9<br>8.7±0.9	4.8–7.6<br>6.0±0.9	6.1–8.9<br>7.5±1.1
Spire height (SpH), mm	2.8–4.7<br>3.5±0.6	2.5–4.3<br>3.4±0.6	3.2–6.3<br>5.0±0.8
Body whorl height (BWH), mm	10.6–12.3<br>11.5±0.5	6.3–9.3<br>7.8±1.1	7.6–12.1<br>9.9±1.5
Aperture height (AH), mm	8.2–11.1<br>9.0±0.8	4.4–6.8<br>5.6±0.7	5.4–8.2<br>6.7±0.9
Aperture width (AW), mm	5.7–8.1<br>6.5±0.7	3.1–5.0<br>4.3±0.7	4.0–6.2<br>5.1±0.8
SW/SH	0.64–0.73<br>0.69±0.03	0.59–0.72<br>0.65±0.03	0.60–0.67<br>0.63±0.02
SpH/SH	0.24–0.33<br>0.28±0.03	0.35–0.41<br>0.37±0.03	0.35–0.46<br>0.42±0.04
BWH/SH	0.90–0.94<br>0.93±0.01	0.83–0.88<br>0.85±0.02	0.83–0.87<br>0.84±0.02
AH/SH	0.66–0.75<br>0.71±0.03	0.57–0.67<br>0.61±0.03	0.55–0.66<br>0.57±0.04
AW/AH	0.68–0.76<br>0.73±0.03	0.62–0.86<br>0.76±0.06	0.67–0.86<br>0.76±0.05

*Only adult snails were selected for measurements.

Supplementary Table 8. Primer sequences for PCR amplification and sequencing

Gene fragment	Primer's name	Direction	Sequence (5'-3')	Reference
COI	LoboF1	Forward	kbtchacaaaycayaargayathgg	Ref. 13
	LoboR1	Reverse	taaacytcwggrtgwccraaraayca	
28S rRNA	D23F	Forward	gagagttcaagagtacgtg	Ref. 14
	D2	Reverse	tccgtgtttcaagacgg	Ref. 15

Supplementary Table 9. List of COI, 16S rRNA and 28S rRNA gene sequences used in phylogenetic reconstruction of the Lymnaeidae. The locality data for the radicine pond snails is given in Supplementary Dataset 1

Genus	Species	Code	COI	16S	28S
Cerasina Kobelt, 1881	*C. luteola* (Lamarck, 1822)	CerLut	JN794496	JN794322	N/A
	C. oxiana (Boettger, 1889)	CerOxy	**MH189935**	JN794336	**MH168046**
	C. siamensis (Sowerby, 1873)	CerSia	**MH190023**	JN794312	**MH168050**
Radix Montfort, 1810	*R. brevicauda* (Sowerby, 1873)	RadBre	JN794435	JN794210	N/A
	R. auricularia (Linnaeus, 1758)	RadAur	**MH189863**	JN794202	**MH168033**
	R. rubiginosa (Michelin, 1831)	RadRub	**MH189925**	N/A	**MH168042**
	R. makhrovi **sp. nov.**	RadMak	**MH189861**	N/A	**MH168032**
	R. plicatula (Benson, 1842)	RadPli	JN794514	JN794348	N/A
	R. alticola (Izzatullaev, Kruglov & Starobogatov, 1983)	RadAlt	**MH189949**	JN794321	**MH168047**
	R. sp. Trichonis	RadTri	EU818805	N/A	N/A
	R. euphratica (Mousson, 1874)	RadEuf	**MH189976**	N/A	**MH168048**
	R. natalensis (Krauss, 1848)	RadNat	EU818835	JN794317	N/A
	R. rufescens (Gray, 1822)	RadRuf	**MH190025**	JN794324	**MH168051**
Ampullaceana Servain, 1881	*A. ampla* (Hartmann, 1821)	AmpAmp	**MH190044**	JN794285	**MH168052**
	A. balthica (Linnaeus, 1758)	AmpBal	**MH189897**	N/A	**MH168039**
	A. intermedia (Lamarck, 1822)	AmpInt	KP242511	N/A	N/A
	A. lagotis (Schrank, 1803)	AmpLag	**MH189858**	JN794342	**MH168030**
	A. relicta (Poliński, 1929)	AmpRel	EU818821	JN794307	N/A
	A. fontinalis (Studer, 1820)	AmpFon	**MH189903**	N/A	**MH168040**
	A. sp. Ohrid	AmpSp1	EU818833	N/A	N/A
	A. cf. *dipkunensis* (Gundrizer & Starobogatov, 1979)	AmpDip	**MH189854**	N/A	**MH168029**
Peregriana Servain, 1881	*P. dolgini* (Gundrizer & Starobogatov, 1979)	PerDol	**MH189886**	N/A	**MH168036**
	P. peregra (O.F. Müller, 1774)	PerPer	**MH189931**	JN794308	**MH168044**
Kamtschaticana Kruglov & Starobogatov, 1984	*K. kamtschatica* (Middendorff, 1851)	KamKam	**MH189919**	N/A	**MH168041**
Myxas G. B. Sowerby I, 1822	*M. glutinosa* (O. F. Müller, 1774)	MyxGlu	EU818798	N/A	N/A
Tibetoradix Bolotov, Vinarski & Aksenova **gen. nov.**	*T. hookeri* (Reeve, 1850) **gen. et comb. nov.**	TibHoo	**MH189865**	JN794204	**MH168034**
	T. kozlovi **sp. nov.**	TibKoz	JN794414	JN794188	N/A
	T. sp.1	TibSp1	JN794395	JN794169	N/A
	T. sp.2	TibSp2	JN794441	JN794216	N/A
	T. sp.3	TibSp3	JN794384	JN794158	N/A
	T. sp.4	TibSp4	JN794436	JN794211	N/A
Orientogalba Kruglov &	*O. ollula* (Gould, 1859)	OriOll	JN794501	JN794327	N/A
	O. cf. *bowelli* (Preston, 1909)	OriBow	JN794473	JN794248	N/A

Genus	Species	Code	COI	16S	28S
Starobogatov, 1985	*O.* cf. *viridis* (Quoy & Gaimard, 1833)	OriVir	**MH189927**	N/A	**MH168043**
Austropeplea Cotton, 1942	*A. tomentosa* (L. Pfeiffer, 1855)	AusTom	AY227365	AF485645	HQ156217
	A. hispida (Ponder & Waterhouse, 1997)	AusHis	N/A	EU556268	N/A
Bullastra Bergh, 1901	*B. lessoni* (Deshayes, 1830)	BulLes	N/A	EU556252	N/A
	B. cumingiana (L. Pfeiffer, 1855)	BulCum	N/A	U82068	N/A
Lymnaea Lamarck, 1799	*L. stagnalis* (Linnaeus, 1758)	LymSta	**MH189887**	U82071	**MH168037**
Kazakhlymnaea Kruglov & Starobogatov, 1984	*K. taurica* (Clessin, 1880)	KazTau	HG932240	N/A	N/A
Stagnicola Jeffreys, 1830	*S. fuscus* (C. Pfeiffer, 1821)	StaFus	HG932234	N/A	N/A
	S. corvus (Gmelin, 1791)	StaCor	**MH189932**	U82079	**MH168045**
	S. palustris (O. F. Müller, 1774)	StaPal	**MH189888**	HQ659900	**MH168038**
Lanx Clessin, 1880	*L. alta* (Tryon, 1865)	LanAlt	HM230361	N/A	HM230318
	L. patelloides (Lea, 1856)	LanPat	HM230363	KT267276	HM230322
Idaholanx Clark, Campbell & Lydeard, 2017	*I. fresti* Clark, Campbell & Lydeard, 2017	IdaFre	HM230356	KT267273	HM230308
Bulimnea Haldeman, 1841	*B. megasoma* (Say, 1824)	BulMeg	N/A	U82069	N/A
Ladislavella B. Dybowski, 1913	*L. catascopium tumrokensis* (Kruglov & Starobogatov, 1985)	LadTum	**KP830103**	N/A	**MH168031**
	L. emarginata (Say, 1821)	LadEma	N/A	U82081	AY465069
	L. liogyra (Westerlund, 1897)	LadLio	**MH190007**	N/A	**MH168049**
	L. terebra (Westerlund, 1885)	LadTer	LT623592	N/A	N/A
	L. occulta (Jackiewicz, 1959)	LadOcc	KP070796	N/A	N/A
	L. bonnevillensis (Call, 1884)	LadBon	N/A	AF485655	N/A
	L. elrodi (Baker & Henderson, 1933)	LadElr	N/A	AF485656	N/A
Sphaerogalba Kruglov & Starobogatov, 1985	*S. bulimoides* (Lea, 1841)	SphBul	EU038362	EU038315	N/A
Galba Schrank, 1803	*G. obrussa* (Say, 1825)	GalObr	N/A	AF485658	HM230317
	G. parva (Lea, 1841)	GalPar	KM612176	N/A	N/A
	G. truncatula (O. F. Müller, 1774)	GalTru	**MH189885**	HQ283236	**MH168035**
	G. neotropica (Bargues, Artigas, Mera y Sierra, Pointier & Mas-Coma, 2007)	GalNeo	KX781342	KX712144	N/A
	G. cubensis (L. Pfeiffer, 1839)	GalCub	FN182205	FN182204	N/A
	G. cousini (Jousseaume, 1887)	GalCou	JN614389	HQ283237	N/A
	G. humilis (Say, 1822)	GalHum	FN182197	FN182195	N/A
	G. schirazensis (Küster, 1862)	GalSch	JF272607	JF272605	N/A
	G. viator (Orbigny, 1835)	GalVia1	JN872451	JN872461	N/A

Genus	Species	Code	COI	16S	28S
	G. viator (Orbigny, 1835)	GalVia2	KT215351	KT215352	N/A
Omphiscola Rafinesque, 1819	*O. glabra* (O. F. Müller, 1774)	OmpGla	FR797862	AY577463	N/A
Aenigmomphiscola Kruglov & Starobogatov, 1981	*A. europaea* Kruglov & Starobogatov, 1981	AenEur	FR797861	N/A	N/A
Pectinidens Pilsbry, 1911	*P. diaphanus* (King, 1832)	PecDia	JN872456	JN872475	N/A
Pseudosuccinea F. C. Baker, 1908	*P. columella* (Say, 1817)	PseCol	KM594623	KM594702	N/A
Fisherola Hannibal, 1912	*F. nuttallii* (Haldeman, 1841)	FisNut	HM230359	HM230355	HM230315
Physella Haldeman, 1843*	*P. acuta* (Draparnaud, 1805)*	PhyAcu	AY651174	AY651213	EF489368
Aplexa J. Fleming, 1820*	*A. elongata* (Say, 1821)*	AplElo	EU038377	EU038330	AY465071

*Outgroup (Physidae). N/A – not available. New sequences generated in this study are in bold.

Supplementary Table 10. Models of sequence evolution for each partition based on corrected Akaike Information Criterion (AICc) of MEGA6 that were applied within a Bayesian inference framework

Partition	Model	Gamma	Invariant
COI			
1st codon of COI	GTR+G	0.47	N/A
2nd codon of COI	TN93+G+I	0.92	0.02
3rd codon of COI	GTR+G+I	0.89	0.55
16S rRNA	GTR+G+I	0.54	0.32
28S rRNA	GTR+G+I	0.64	0.52

N/A – not available.

Supplementary Table 11. List of fossil and tectonic calibrations that were used in BEAST analyses

Calibration no.	Calibration Type	MRCA	Description	Reference
Calibration 1	Fossil record	Lymnaeidae	Hard minimum age: 152.1 Ma, †*Lymnaea morrisonensis* Yen (1952): Pl. 6, Figs. 19A-19B[16]. Diagnosis and phylogenetic placement: Shell of moderate size, thin substance, ovately oblong outline, having a subconical and highly elevated spire and descending dilated body whorl, the spire smaller than the body whorl. Whorls increasing rapidly in size, gently convex, slightly shouldered below the suture and bearing distinct lines of growth. Aperture subovate in outline, outer lip thin and simple, parietal margin attenuated but well defined, and columella slightly twisted at its upper part, with its margin reflected and attached firmly over the umbilical area[16]. The fossil records from the Morrison Formation are considered the earliest known lymnaeids[17] but it might also be a steam lineage of the Lymnaeidae. We therefore placed this fossil on the root node of the family as a stem calibration. Stratigraphic horizon and locality: Morrison Formation, Felch's Ranch, Garden Park, 9 miles north of Canon City, Fremont County, Colorado[16]. Absolute age estimate: Late Jurassic, Kimmeridgian/Tithonian boundary, 152.1 Ma, based on stratigraphy[17]; 95% soft upper bound 157.3 Ma based on the lower Kimmeridgian boundary. Prior setting: exponential distribution, mean (lambda) = 1.4, MRCA: *Omphiscola glabra* – *Radix makhrovi* sp. nov.	This study: New stem calibration
Calibration 2	Tectonic event	*Tibetoradix*	Hard minimum age: 14.9 Ma, tectonic event, the start of the second phase of tectonic uplift of the Tibetan Plateau[18]. Phylogenetic placement: We placed this tectonic event as a crown calibration for *Tibetoradix* that is considered an endemic Tibetan clade. Stratigraphic horizon and locality: Xia Ganchaigou Formation, China[18]. Absolute age estimate: Middle Miocene, 14.9 Ma, Shang Youshashan Formation, based on stratigraphic and magnetostratigraphic correlations; 95% soft upper bound 31.5 Ma based on the peak of the first phase of tectonic uplift of the Tibetan Plateau in the Early Oligocene[18]. Prior setting: exponential distribution, mean (lambda) = 4.5, MRCA: *Tibetoradix* sp.1 – *Tibetoradix* sp.4.	This study: New crown calibration

Calibration no.	Calibration Type	MRCA	Description	Reference
Calibration 3	Fossil record	*Radix* s. str. (*R. alticola* group + *R. auricularia* group)	Hard minimum age: 16.5 Ma, †*Radix socialis* (von Zieten, 1832) [Salvador & Rasser (2014): Fig. 10][19]<br>Diagnosis and phylogenetic placement: Shell small, rounded, *Radix*-like form; shell width ca. 2/3 shell height. Protoconch rounded, smooth; transition to teleoconch unclear. Teleoconch smooth, except for growth lines. Sulture deep, well-marked. Whorls profile markedly convex. Aperture apparently oval, elongated. Peristome apparently simple, sharp. Umbilicus apparently imperforate[19]. This fossil species appears to be an early member of the *R. alticola* group + *R. auricularia* group subclade and it placed by us as a crown calibration.<br>Stratigraphic horizon and locality: Early/Middle Miocene, Sandelzhausen fossil site, near Mainburg, Molasse Basin, southern Germany[19].<br>Absolute age estimate: Middle Miocene, near the Burdigalian/Langhian boundary, 16.5 Ma, based on stratigraphic, biostratigraphic, and magnetostratigraphic correlations[19]; 95% soft upper bound 33.0 Ma (twice the age of the fossil).<br>Prior setting: exponential distribution, mean (lambda) = 4.5, MRCA: *Radix alticola* – *R. auricularia*.	This study: New crown calibration
Calibration 4	Fossil record	*Ampullaceana*	Hard minimum age: 16.3 Ma, †*Radix enzenbachensis* Neubauer & Harzhauser (2014): Pl. 2, Figs. 1–3[20].<br>Diagnosis and phylogenetic placement: Broad globose shell with short spire and inflated and highly convex last whorl; aperture expanded laterally, with strong angulation between straight columella and inner lip[20]. This fossil species appears to be an early member of the *Ampullaceana* clade and it placed by us as a crown calibration.<br>Stratigraphic horizon and locality: Lower Badenian lacustrine marls of the Rein beds, Enzenbach, Rein Basin, Styria, Austria[20].<br>Absolute age estimate: Middle Miocene, Lower Badenian, 16.3 Ma, based on stratigraphic, magnetostratigraphic, climatic and sea-level changes correlations in addition to isotope events and biostratigraphic data[21]; 95% soft upper bound 32.6 Ma (twice the age of the fossil).<br>Prior setting: exponential distribution, mean (lambda) = 4.4, MRCA: *Ampullaceana ampla* – *A. balthica*	This study: New crown calibration
Calibration 5	Fossil record	*Peregriana*	Hard minimum age: 12.5 Ma, †*Radix hyaloleuca* (Brusina, 1902) [Kókay (2006): Pl. 17, Fig. 15][22].<br>Diagnosis and phylogenetic placement: With	This study: New stem calibration

Calibration no.	Calibration Type	MRCA	Description	Reference
			respect to the image of a well-preserved fossil shell[22], this fossil species may represent an early steam lineage of the *Peregriana* clade and it placed by us as a stem calibration.<br>Stratigraphic horizon and locality: Sarmatian of Várpalota, Hungary[22].<br>Absolute age estimate: Middle Miocene, Sarmatian, 12.5 Ma, based on integrative stratigraphic correlations[23]; 95% soft upper bound 25.0 Ma (twice the age of the fossil).<br>Prior setting: exponential distribution, mean (lambda) = 3.4, MRCA: *Peregriana labiata – P. dolgini*	
Calibration 6	Fossil record	*Stagnicola*	Hard minimum age: 18.5 Ma, †*Stagnicola subpalustris* (Thomä, 1845) [Harzhauser et al. (2014): Figs. 3D-3E][24].<br>Diagnosis and phylogenetic placement: With respect to the image of a well-preserved fossil shell[24], this fossil species appears to be an early member of the *Stagnicola* clade and it placed by us as a crown calibration.<br>Stratigraphic horizon and locality: Korozluky, Tuchořice, Pyšná, and Lipno in the North Bohemian Lake, the Most Basin, Czech Republic[24].<br>Absolute age estimate: Early Burdigalian, 18.5 Ma, based on stratigraphic correlations[24]; 95% soft upper bound 37.0 Ma (twice the age of the fossil).<br>Prior setting: exponential distribution, mean (lambda) = 5.0, MRCA: *Stagnicola fuscus – S. palustris*.	This study: New crown calibration
Calibration 7	Fossil record	*Galba*	Hard minimum age: 16.5 Ma, †*Galba dupuyiana* (Noulet, 1854) [Salvador & Rasser (2014): Figs. 1-7][19].<br>Diagnosis and phylogenetic placement: Shell height 5-7 mm, shell width ca. 1/2 shell height, 4-4.5 whorls, protoconch rounded, smooth, transition to teleoconch unclear, teleoconch smooth, except for well-marked growth lines. Suture deep, well-marked. Whorls profile convex. Aperture oval, narrow, elongated, ca. 2/3 shell height. Peristome simple, almost completely covering umbilicus. Umbilicus rimate. With respect to the images of well-preserved fossil shells[19], this fossil species appears to be an early member of the *Galba* clade and it placed by us as a crown calibration.<br>Stratigraphic horizon and locality: Early/Middle Miocene, Sandelzhausen fossil site, near Mainburg, Molasse Basin, southern Germany[19].<br>Absolute age estimate: Middle Miocene, near the Burdigalian/Langhian boundary, 16.5 Ma, based	This study: New crown calibration

Calibration no.	Calibration Type	MRCA	Description	Reference
			on stratigraphic, biostratigraphic, and magnetostratigraphic correlations[19]; 95% soft upper bound 33.0 Ma (twice the age of the fossil). Prior setting: exponential distribution, mean (lambda) = 4.5, MRCA: *Galba schirazensis* – *G. parva*.	

Supplementary Table 12. Evaluation of calibration lineages* based on the empirical scaling factor (ESF)[25]

**Calibration No.**	**MRCA**	**Node-to-tip length relative to base of tree (*NTL_i*)**	**Calibration, Ma (*FA_i*)**	**Empirical scaling factor (*$si = FA_i / NTL_i$*)**
Calibration 1	Lymnaeidae	1.0000	152.1	152
Calibration 2	*Tibetoradix*	0.1672	14.9	89
Calibration 3	*Radix* s. str. (*R. alticola* group + *R. auricularia* group)	0.1951	16.5	85
Calibration 4	*Ampullaceana*	0.1864	16.3	87
Calibration 5	*Peregriana*	0.2226	12.5	56
Calibration 6	*Stagnicola*	0.2075	18.5	89
Calibration 7	*Galba*	0.3603	16.5	46

*Supplementary Table 11.

Supplementary Note. Systematic list of the Old World radicine taxa recovered during our study, with notes on their taxonomic position and nomenclature

This list provides brief accounts of the Old World radicine taxa accepted as valid in our study except for six species-level clades, i.e. *Radix* sp. Trichonis, *Ampullaceana* sp. Ohrid, *Tibetoradix* sp.1, *T.* sp.2, *T.* sp.3, and *T.* sp.4, the available names of which were not detected by us. We provide the data on their original descriptions, type series (if available), and *loci typici*. In some cases, the taxonomic and nomenclatural notes are added. The full taxonomic account for these taxa, with illustrations of type specimens, measurements, diagnostic remarks and other information needed for their identification will be published elsewhere as a separate article.

Genus *Radix* Montfort, 1810

Subgenus *Radix* s. str.

Radix Montfort, 1810: 266[26].

1. *Radix* (*Radix*) *auricularia* (Linnaeus, 1758)

Helix auricularia Linnaeus, 1758: 774-775[27].

Type locality: Europe.

Types: Possibly lost[28].

This species has many times been characterized in the literature, both conchologically and anatomically. The specimens of *R. auricularia* studied by us corresponded to the commonly accepted views on the identity of this species[11,29,30].

2. *Radix* (*Radix*) *alticola* (Izzatullaev, Kruglov et Starobogatov, 1983)

Lymnaea (*Radix*) *alticola* Izzatullaev *et al*., 1983: 53, fig. 1-2[31].

Type locality: Tajikistan, a hot spring near the Yashilkul' Lake (approximately 37°47'00"N; 72°51'00"E).

Types: ZISP[28]. The holotype is illustrated by Sitnikova *et al*.[32]. We studied both the holotype and the paratypes of *R. alticola*.

We examined specimens of *R. alticola* collected in mountain Tajikistan (Pamir). Their morphology was fully consistent with that of the type specimens.

3. *Radix* (*Radix*) *brevicauda* (G.B. Sowerby II, 1873)

Limnaea brevicauda G.B. Sowerby II, 1873: pl. XV, fig. 105[33].

Type locality: The type locality was originally stated as 'Australia'[33]. It is, however, erroneous. Hanley & Theobald[34] gave the proper type locality: Kashmir.

Types: NHMUK. The syntypes were inspected by us.

Nomenclature remark. The name *Limnaea brevicauda* Sowerby is the oldest available one to designate a lymnaeid species, sister to *R. auricularia*, restricted in its distribution to the Central Asian mountain regions. The taxonomic identity of *R. brevicauda* as well its close affinity to *R. auricularia* were confirmed by the inspection of the extant syntypes.

4. *Radix* (*Radix*) *euphratica* (Mousson, 1874)

Limnaea euphratica Mousson, 1874: 40-41[35].

Type locality: Iraq, vicinity of Es-Samava Town (approximately 31°19´00˝N; 45°17´00˝E).

Types: Not traced, but probably in the Zürich Zoological Miseum[28].

Nomenclature remark. Several nominal species of radicines, with type localities situated in the Middle East or the east of Central Asia, were described in the late 19th – first half of the 20th century. The oldest of them are *Limnaeus tener* Küster, 1862, *Limnaea auricularia* var. *persica* Bourguignat in Issel, 1865, and *Limnaea euphratica* Mousson, 1874.

The types of *L. tener* are lost, while the holotype of *L. auricularia* var. *persica* is extant[36]. Though the type series of *R. euphratica* is most probably lost, the shells of this species collected in Iraq (NHMUK) look like shells of snails from Tajikistan studied by us both genetically and morphologically. It allowed us to choose the name *Limnaea euphratica* Mousson, 1874 for the designation of this species, since the shell habitus of both *L. tener* and *L. auricularia* var. *persica* is different from that of snails from Iraq.

5. ***Radix*** **(*Radix*)** ***makhrovi*** **Bolotov, Vinarski & Aksenova** sp. n.

Type locality: China, Tibet, a roadside ditch west of the Lhasa River mouth, Brahmaputra River basin.

Types: ZISP (holotype, paratypes), RMBH (paratypes).

6. ***Radix*** **(*Radix*)** ***plicatula*** **(Benson in Cantor, 1842)**

Limnaea plicatula Cantor, 1842: 487[37].

Taxonomic remark. *Radix plicatula* is almost indistinguishable from *R. euphratica* by its shell morphology and the copulatory organ structure, but the molecular analysis have revealed that the two species are distinct and their ranges are almost non-overlapping. There is a small series of shells of this species, collected from the type locality, in NHMUK (accession No. 42.9.30.50.51.487). These apparently once belonged to Benson's collection and may be considered the probable syntypes. Their morphology is very similar to that in *R. plicatula* snails examined genetically by us.

7. ***Radix*** **(*Radix*)** ***rubiginosa*** **(Michelin, 1831)**

Limnoeus rubiginosus Michelin, 1831: 22[38].

Type locality: The East-Indies. "The original specimens are said to come from Bogor in Java" (Brandt, 1974: 230[39]).

Types: Probably lost. We failed to find them in NMNH collection.

This southeast Asian species is relatively well-studied and the views on its identity expressed by most authors are in agreement with each other[4,39,40].

Subgenus *Exsertiana* Bourguignat, 1883

Exsertiana Bourguignat, 1883: 88[41].

Nomenclature remark. Bourguignat (P. 88)[41], in his article devoted to classification of continental molluscs of Abyssinia, established two new groups of species within the genus *Limnaea* – *Exsertiana* and *Raffrayana*. He did not give any diagnosis for the two taxa, only lists of species included there. In both cases, these species are currently recognized as junior synonyms of *R. natalensis*[4,42]. Acting as the First Revisers, we select *Exsertiana* as a name for the designation of a lymnaeid clade containing *R. natalensis*.

8. *Radix* (*Exsertiana*) *natalensis* (Krauss, 1848)

Limnaeus natalensis Krauss, 1848: 85[43].

Type locality: South Africa, Natal.

Types: Probably lost. We failed to find them either in ZMB collection or in other institutions (NMNH, NHMUK) known to contain vast collections of African aquatic snails.

R. natalensis is a species widely distributed in Africa; its identity is based on a morphological data[42] as well as on the results of molecular works (Ref. 44; this study).

9. *Radix* (*Exsertiana*) *rufescens* (Gray in Sowerby, 1822)

Lymnaea acuminata Lamarck, 1822: 160[45] (invalid; a junior homonym of *Lymnaea acuminata* Brogniart, 1810).

Limnaea rufescens Sowerby, 1822: 44, pl. 178, fig. 2[46].

Type locality: "the East Indies".

Types: Probably lost.

The specimens of *R. rufescens* studied by us molecularly corresponded to the morphological descriptions of this species available from the literature[4,47,48].

The next three genera (*Ampullaceana*, *Kamtschaticana*, and *Peregriana*) represent parts of the former subgenus *Peregriana* s. lato of the genus *Lymnaea*[8,11], or *Radix*[12,28].

Genus *Ampullaceana* Servain, 1881

Ampullaceana Servain, 1881: 53[49].

10. *Ampullaceana ampla* (W. Hartmann, 1821)

Limneus auricularia var. *ampla* Hartmann, 1821: 250, Taf. II, fig. 29[50].

Type locality: Germany, Bavaria, Rhein River near Reineck.

Lectotype: Naturmuseum Saint-Gallen, Switzerland (designated in Vinarski & Glöer[51]).

This species is well-studied. It has repeatedly been characterized in the recent literature under the name *Radix* ampla[29,51,52].

11a. *Ampullaceana relicta relicta* (Poliński, 1929)

Radix relicta Poliński, 1929: 158[53].

Type locality: Lake Ohrid.

Types: Whereabouts unknown.

See Albrecht *et al.*[54] and Welter-Schultes[52] for morphological and molecular characterization of this taxon.

11b. *Ampullaceana relicta pinteri* (Schütt, 1974)

Radix pinteri Schütt, 1974: 471[55].

Type locality: Macedonia, Lake Prespa near village of Perovo.

Types: Whereabouts unknown.

See Albrecht *et al*.[54] and Welter-Schultes[52] for morphological and molecular characterization of this taxon.

12. *Ampullaceana dipkunensis* (Gundrizer et Starobogatov, 1979)

Lymnaea dipkunensis Gundrizer & Starobogatov, 1979: 1134, fig. 1 (4)[56].

Lymnaea (*Peregriana*) *tumida* Kruglov & Starobogatov, 1993: 166, fig. 6 G, non Held, 1836[8].

Type locality: Russia, Krasnoyarsk Territory, Gornoye Lake in the floodplain of the Kureika River upstream of the mouth of the Dipkun River.

Types: ZISP (examined by us).

Taxonomic remark. The snails studied by us molecularly may be identified with the species *Lymnaea* (*Peregriana*) *tumida* (Held, 1836) sensu Kruglov & Starobogatov, 1993. However, to use this species name is hardly acceptable. This taxon is usually considered as a intraspecific morph of *Radix auricularia* living in large Alpine lakes[29,57]. The examination of the topotypes (dried shells) of *L. tumida* kept in different European museums (NHMW, NMG, ZMUC) allowed us to agree with this opinion. Therefore we used the next oldest available name, *Lymnaea dipkunensis*, to designate this clade. The identity of this taxon was revealed by means of the inspection of the type series (see also Ref. 32). The record of *Lymnaea tumida* sensu Kruglov & Starobogatov in the Lower Yenissei Basin[58], the type region of *L. dipkunensis* gives an indirect evidence in favor of their identity.

13. *Ampullaceana lagotis* (Schrank, 1803)

Buccinum lagotis Schrank, 1803: 290[59].

Type locality: Germany, Bavaria.

Types: Lost.

The species has been characterized both genetically and morphologically[11,29,60]. Individuals of *A. lagotis* used in this studied were similar to specimens of this species described in Ref. 60.

14. *Ampullaceana fontinalis* (Studer, 1820)

Limneus fontinalis Studer, 1820: 93[61].

Type locality: Switzerland.

Lectotype: Naturhistorisches Museum der Burgergemeinde Bern, Switzerland.

Taxonomic remark. Specimens of this species-rank clade studied by us were identical to snails designated by Kruglov & Starobogatov[8] as *Lymnaea* (*Peregriana*) *fontinalis*. The shells of *L. fontinalis* sensu Kruglov & Starobogatov[8] are similar to the lectotype shell illustrated by Forcart[62]. This species is morphologically close to *A. lagotis* but may be distinguished by a lower spire and more inflated body whorl.

15. *Ampullaceana balthica* (Linnaeus, 1758)

Helix balthica Linnaeus, 1758: 775[27].

Type locality: Sweden, Baltic Sea shore[27]. The locality of the neotype is "Sweden, Stockholm"[63].

Neotype: ZISP (designated by Kruglov & Starobogatov[63]).

The concept of this species accepted here coincides with that of Glöer[29] and Schniebs *et al.*[64].

16. *Ampullaceana intermedia* (Lamarck, 1822)

Lymnaea intermedia Lamarck, 1822: 162[45].

Type locality: France, Quercy Plateau.

Types: Probably, Muséum d'Histoire Naturelle, Genève, Switzerland.

We applied the binomen *Lymnaea intermedia* as the oldest available name for designation of a species sister to *A. balthica*, which is widely distributed in France and adjacent countries. Kruglov & Starobogatov[8,63] also used this binomen to name a species occurring throughout Northern Palearctic, however, a special study is needed to see whether the concept proposed by Kruglov & Starobogatov[8,63] is applicalble to *L. intermedia* sensu Lamarck, 1822[45].

Genus *Peregriana* Servain, 1881

Peregriana Servain, 1881: 56[49].

17. *Peregriana peregra* (O.F. Müller, 1774)

Buccinum peregrum O.F. Müller, 1774: 130[65].

Radix labiata Falkner *et al*., 2002: 94[66].

Type locality: Denmark, Copenhagen, Frederiksberg Park, in swamps[28].

Types: Lost[67].

For morphological and molecular characterization of this species see Schniebs *et al*.[68] and Vinarski *et al*.[69].

Nomenclature remark. Falkner *et al.*[66] argued that the lymnaeid species, which had been commonly named as *Radix* (or, *Lymnaea*) *peregra* by the European authors, does not occur in Denmark, the type country of Müller's *Buccinum peregrum*. These authors treated *B. peregrum* as a junior synonym of *Helix balthica* Linnaeus, and proposed a name *Radix labiata* (Rossmässler, 1835) for designation of *R. peregra* auct. Such authors as Glöer[29] and Welter-Schultes[52] followed it, however Vinarski[70] doubted this decision. According to him, there is no the total evidence of the absence of *R. peregra* in Denmark, whereas the type specimens of *Limnaeus pereger* var. *labiatus* (kept in NHMW) were considered by Vinarski[70] as juvenile individuals of *R. balthica*.

18. *Peregriana dolgini* (Gundrizer et Starobogatov, 1979)

Lymnaea dolgini Gundrizer & Starobogatov, 1979: 1132, fig. 1(2); 2 (2)[56].

Type locality: Russia, Krasnoyarsk Territory, a lake in the floodplain of the Kureika River, 20 km upstream of its mouth.

Types: ZISP.

For morphological and molecular characterization of this species see our previous article[69].

Genus *Kamtschaticana* Kruglov et Starobogatov, 1984

Kamtschaticana Kruglov & Starobogatov, 1984: 30[71].

19. *Kamtschaticana kamtschatica* (Middendorff, 1851)

Limnaeus kamtschaticus Middendorff, 1851: 295, pl. 30, fig. 11-12[72].

Type locality: Russia, Kamchatka Peninsula, Kamchatka River (Kruglov & Starobogatov[71] suggest that the type specimens were collected from the floodplain of the river).

Lectotype: ZISP (see Ref. 71 for details).

For morphological and molecular characteristics of this species see our previous article[73].

Genus *Myxas* G. B. Sowerby I, 1822

Myxas G.B. Sowerby, 1822: part vii[46].

20. *Myxas glutinosa* (O.F. Müller, 1774)

Buccinum glutinosum O.F. Müller, 1774: 129[65].

Type locality: Not stated in the original description. Most probably, the type locality should be quoted as Fridrichsdal, a suburb of Copenhagen, Denmark (see Ref. 67 for details).

Types: Lost[67].

The taxonomic position and identity of this morphologically peculiar species have not risen much doubts, and most authors treated it more or less identically[4,29,52] (but see Kruglov & Starobogatov[8]). The results of our study well correspond to the commonly accepted concept of *M. glutinosa*.

Genus *Tibetoradix* Bolotov, Vinarski & Aksenova **gen. nov**

21. *Tibetoradix hookeri* (Reeve, 1850)

Lymnaea hookeri Reeve, 1850: 49[74].

Type locality: "Thibetan or north side of Sikkim Himalaya, at 18,000 feet elevation".

Types: NHMUK (examined by us).

The snails studied by us molecularly were conchologically almost identical to the syntypes of *Lymnaea hookeri* Reeve kept in NHMUK.

Taxonomic remark. Kruglov & Starobogatov[8] classified this species as belonging to the (sub)genus *Orientogalba* and as a senior synonym of *Lymnaea heptapotamica* Lazareva, 1967, described from southern Kazakhstan. The examination of the syntypes of *L. hookeri* has shown that *L. heptapotamica* should not be synonymized with the former species, and may well represent a separate taxon.

22. *Tibetoradix kozlovi* Vinarski, Bolotov & Aksenova sp. n.

Type locality: China, Central Tibet, the floodplain of the Requ Qu River, Yellow River basin, 33°35'20.7" N, 103°05'30.2" E, alt. 3,470 m.

Types: ZISP (holotype, paratypes), RMBH (paratypes).

Genus *Cerasina* Kobelt, 1881

Cerasina Kobelt, 1881: 297[75].

23. *Cerasina luteola* (Lamarck, 1822)

Lymnaea luteola Lamarck, 1822: 160[45].

Type locality: India, Bengalia.

Types: Not traced, probably in Muséum d'Histoire Naturelle, Genève, Switzerland.

Most authors[4,39,47] accept this species in a broad sense, as a single member of *Cerasina*. During this study, we recovered at least three separate species in this group. One of them, distributed in India and Nepal has been identified with the Lamarck's species.

24. *Cerasina oxiana* (Boettger, 1889)

Limnaeus impurus var. oxiana Boettger, 1889: 961, Taf. 27, fig. 4-5[76].

Type locality: Turkmenistan, middle Amu-Darya River, 'Tschardschui' (nowadays Türkmenabat).

Types: Not traced.

This species of *Cerasina* is distributed in Central Asia (within the ex-USSR boundaries) and Nepal. The oldest available name, based on a type locality situated within this area, is *C. oxiana*. Though Kruglov & Starobogatov[8] treated this taxon as identical with *Cerasina impura* (Troschel, 1837), the latter species has its type locality in India, where *C. oxiana* does not occur. *Cerasina impura* is, most probably, a junior synonym of *C. luteola*[4,39].

25. *Cerasina siamensis* (G.B. Sowerby II, 1873)

Limnaea siamensis G.B. Sowerby II, 1873: pl. X, fig. 63[33].

Type locality: Thailand (Siam).

Types: Not traced.

We used this name as an oldest one applicable to a species of *Cerasina*, which is distributed in Myanmar and Thailand. Though Brandt[39] considered *L. siamensis* as a junior synonym of *R. rubiginosa*, our data show it is untenable.

Genus *Orientogalba* Kruglov et Starobogatov, 1985

Orientogalba Kruglov & Starobogatov, 1985: 28[77].

This lymnaeid genus needs a thorough revision. According to our results, it includes not less than three biological species, whose nomenclature and distribution have to be clarified. In our opinion, this genus should not be united with the genus *Austropeplea* Cotton, 1942, as it was suggested by some authors[78,79].

26. *Orientogalba viridis* (Quoy et Gaimard, 1832)

Lymnaea viridis Quoy & Gaimard, 1832: 204, pl. 58, figs. 16-18[80].

Type locality: The Pacific, Marian Archipelago, Guam Island.

Syntypes: MNHN (examined by us).

Our concept of this species is based on the type series of *O. viridis* (see Ref. 79 for illustrations of the syntypes).

27. *Orientogalba ollula* (Gould, 1859)

Limnaea ollula Gould, 1859: 40[81].

Type locality: China, streams and marshes of Hong Kong Island.

Lectotype: National Museum of Natural History, Smithsonian Institution, Washington, USA[82].

Taxonomic remark. This species, described from eastern China, has usually been considered as a junior synonym of *O. viridis*[4,39], however Kruglov & Starobogatov[8] regarded it as a separate species. The results of our study allowed us to accept their opinion and use

the name *O. ollula* to label a species of *Orientogalba*, widely distributed in China (found also in South Korea and Nepal).

28. *Orientogalba* cf. *bowelli* (Preston, 1909)

Limnaea bowelli Preston, 1909: 115, fig. 1[83].

Type locality: Tibet, "Te-ring Gompa, in a small hill stream arising from a spring, 14,000 feet; also from Mangtsa, 14,500 feet; High Hill, Gompa, Gyantse valley in a small hill stream, among moss and stones, 14,500 feet; and Gyantse, 13,120 feet".

Types: Zoological Survey of India, Kolkata (fide Ref. 47).

The application of the binomen *Limnaea bowelli* Preston is difficult. Recent authors tend to consider it as a member of the (sub)genus *Galba*, whose distribution is restricted to the High Asia[11,28,47]. On the other hand, Hubendick[4] hypothesized that *Limnaea bowelli* is identical with *Radix auricularia*. After having examined several small samples of this species kept in NHMUK and NHMN, we found that these shells may belong to *Orientogalba*; some of them are very similar to shells of *O. viridis*. The type series was unavailable for us, and here we conditionally attach the Preston's taxon name to a species of *Orientogalba* found in Sichuan Province of China.

Genus *Bullastra* Bergh, 1901

Bullastra Bergh, 1901: 254[84].

29. *Bullastra cumingiana* (L. Pfeiffer, 1845)

Amphipeplea cumingiana Pfeiffer 1845: 68[85].

Type locality: Philippines, island of Luzon, Naga, province of South Camerines.

Probable syntypes: ZMB (examined by us).

Hubendick[4] gave an illustrated morphological description of this strictly Philippinian lymnaeid species. Its identity is clear from the (probable) syntypes studied and illustrated by Vinarski[86] (2016).

Supplementary References

1. Pfenninger, M., Cordellier, M. & Streit, B. Comparing the efficacy of morphologic and DNA-based taxonomy in the freshwater gastropod genus *Radix* (Basommatophora, Pulmonata). *BMC Evolutionary Biology* **6**, 100; DOI:10.1186/1471-2148-6-100 (2006).
2. von Oheimb, P. V. et al. Freshwater biogeography and limnological evolution of the Tibetan Plateau – Insights from a plateau-wide distributed gastropod taxon (*Radix* spp.). *PLoS ONE* **6,** e26307; DOI:10.1371/journal.pone.0026307 (2011).
3. Clewing, C., Albrecht, C. & Wilke, T. A complex system of glacial sub-refugia drives endemic freshwater biodiversity on the Tibetan Plateau. *PLoS ONE* **11**, e0160286; DOI:10.1371/journal.pone.0160286 (2016).
4. Hubendick, B. Recent Lymnaeidae. Their variation, morphology, taxonomy, nomenclature and distribution. *Kungliga Svenska Vetenskapsakademiens Handlingar. Fjärde Serien* **3**, 1–223 (1951).
5. Correa, A. C. et al. Bridging gaps in the molecular phylogeny of the Lymnaeidae (Gastropoda: Pulmonata), vectors of fascioliasis. *BMC Evolutionary Biology* **10**, 381; DOI:10.1186/1471-2148-10-381 (2010).
6. Patterson, C. M. & Burch, J. B. Chromosomes of pulmonate molluscs. in Fretter, V. & Peake, J. (eds.). *Pulmonates. Vol. 2A.* 171–217 (London, 1978).
7. Kruglov, N. D. & Starobogatov, Y. I. Annotated and illustrated catalogue of species of the family Lymnaeidae (Gastropoda Pulmonata Lymnaeiformes) of Palaearctic and adjacent river drainage areas. Part I. *Ruthenica* **3**, 65–92 (1993).
8. Kruglov, N. D. & Starobogatov, Y. I. Annotated and illustrated catalogue of species of the family Lymnaeidae (Gastropoda Pulmonata Lymnaeiformes) of Palaearctic and adjacent river drainage areas. Part 2. *Ruthenica* **3**, 161–180 (1993).
9. Kruglov, N. D. & Starobogatov, Y. I. *Myxas*-similar Lymnaeidae (Gastropoda, Pulmonata), their origin and specific composition. *Byulleten' Moskovskogo Obshchestva Ispytateley Prirody, Otdel Biologicheskiy* **90**, 69–78 (1985).
10. Ponder, W. F. & Waterhouse, J. A new genus and species of Lymnaeidae from the lower Franklin River, south western Tasmania. *Journal of Molluscan Studies* **63**, 441–468; DOI:10.1093/mollus/63.3.441 (1997).
11. Kruglov, N. D. *Mollusks of the family Lymnaeidae (Gastropoda Pulmonata) in Europe and northern Asia* (Smolensk, 2005).
12. Vinarski, M. V. One, two, or several? How many lymnaeid genera are there? *Ruthenica* **23**, 41–58 (2013).
13. Lobo, J. et al. Enhanced primers for amplification of DNA barcodes from a broad range of marine metazoans. *BMC Ecology* **13**, 34; DOI:10.1186/1472-6785-13-34 (2013).

14. Park, J.-K. & Ó Foighil, D. Sphaeriid and corbiculid clams represent separate heterodont bivalve radiations into freshwater environments. *Molecular Phylogenetics and Evolution* **14**, 75–88; DOI:10.1006/mpev.1999.0691 (2000).
15. Jovelin, R. & Justine, J. L. Phylogenetic relationships within the polyopisthocotylean monogeneans (Platyhelminthes) inferred from partial 28S rDNA sequences. *International Journal for Parasitology* **31**, 393–401; DOI:10.1016/S0020-7519(01)00114-X (2001).
16. Yen, T.-C. Molluscan fauna of the Morrison Formation. *Geological Survey Professional Paper* **233,** 21–51 (1952).
17. Gray, J. Evolution of the freshwater ecosystem: the fossil record. *Palaeogeography, Palaeoclimatology, Palaeoecology* **62**, 1–214; DOI:10.1016/0031-0182(88)90054-5 (1988).
18. Wang, Y. et al. Cenozoic uplift of the Tibetan Plateau: Evidence from the tectonic–sedimentary evolution of the western Qaidam Basin. *Geoscience Frontiers* **3**, 175–187; DOI:10.1016/j.gsf.2011.11.005 (2012).
19. Salvador, R. B. & Rasser, M. W. The fossil pulmonate snails of Sandelzhausen (Early/Middle Miocene, Germany) (Hygrophila, Punctoidea and limacoids). *Archiv für Molluskenkunde: International Journal of Malacology* **143**, 187–202; DOI:10.1127/arch.moll/1869-0963/143/187-202 (2014).
20. Harzhauser, M., Neubauer, T. A., Gross, M. & Binder, H. The early Middle Miocene mollusc fauna of Lake Rein (Eastern Alps, Austria). *Palaeontographica A* **302**, 1–71 (2014).
21. Hohenegger, J., Ćorić, S. & Wagreich, M. Timing of the middle Miocene Badenian stage of the central Paratethys. *Geologica Carpathica* **65**, 55–66; DOI:10.2478/geoca-2014-0004 (2014).
22. Kókay, J. Nonmarine mollusc fauna from the Lower and Middle Miocene, Bakony Mts, W Hungary. *Geologica Hungarica. Ser. Palaeontologica* **56**, 1–196 (2006).
23. Harzhauser, M. & Piller, W. E. Integrated stratigraphy of the Sarmatian (Upper Middle Miocene) in the western Central Paratethys. *Stratigraphy* **1**, 65–86 (2004).
24. Harzhauser, M., Neubauer, T. A., Georgopoulou, E. & Harl, J. The Early Miocene (Burdigalian) mollusc fauna of the North Bohemian Lake (Most Basin). *Bulletin of Geosciences* **89**, 819–908 (2014).
25. Marshall, C. R. A simple method for bracketing absolute divergence times on molecular phylogenies using multiple fossil calibration points. *The American Naturalist* **171,** 726–742; DOI:10.1086/587523 (2008).
26. Montfort, P. D. de. *Conchyliologie Systématique et Classification Méthodique des Coquilles. Vol. 2: Coquilles univalves, non cloisonées* (Paris, 1810).
27. Linnaeus, C. *Caroli Linnaei ... systema naturae per regne tria naturae, secundum classes, ordines, genera, species, cum characteribus, differentiis, synonymis, locis... Editio decima reformata* (Holmiae, 1758).
28. Vinarski, M. V. & Kantor, Yu. I. *Analytical catalogue of fresh and brackish water molluscs of Russia and adjacent countries* (Moscow, 2016).

29. Glöer, P. *Die Sußwassergastropoden Nord- und Mitteleuropas: Bestimmungschlussel, Lebenweise, Verbreitung* (Hackenheim, 2002).

30. Jackiewicz, M. European species of the family Lymnaeidae (Gastropoda, Pulmonata, Basommatophora). *Genus* **9**, 1–93 (1998).

31. Izzatullaev, Z.I., Kruglov, N.D. & Starobogatov, Ya.I. New and ill-known species of mollusks of the subgenus *Radix* of the genus *Lymnaea* of the USSR fauna from the Central Asia (Gastropoda, Pulmonata) [In Russian]. *Izvestiya Akademii Nauk Tajikskoy SSR, Otdelenie biologicheskikh nauk* **4,** 53–57 (1983).

32. Sitnikova, T.Ya., Sysoev, A.V. & Prozorova, L.A. Types of freshwater gastropods described by Ya.I. Starobogatov, with additional data on the species: family Lymnaeidae. *Zoologicheskie Issledovaniya* **16**, 7–37 (2014).

33. Sowerby, G.B. Monograph of the genus *Limnaea*. *Conchologia Iconica* **18**, no pagination, plates 1–15 (1873).

34. Hanley, S. & Theobald, W. *Conchologia Indica: Illustrations of the Land and Freshwater Shells of British India* (London, 1876).

35. Mousson, A. Coquilles terrestres et fluviatiles recueillies par M. le Dr. Alex Schlaefli dans l'Orient. *Journal de Conchyliologie* **22**, 5–60 (1874).

36. Sitnikova, T.Ya., Kijashko, P.V. & Sysoev, A.V., Species names of J.-R. Bourguignat and their application in current taxonomy of fresh-water gastropods of the Russian fauna. *Bulletin of the Russian Far East Malacological Society* **15-16**, 87–116 (2012).

37. Cantor, T. General features of Chusan, with remarks on the flora and fauna of that island. *Annals and Magazine of Natural History (series 1)* **9**, 486–489 (1842).

38. Michelin H. *Lymnoeus rubiginosus*. *Magasin de Zoologie*. *Première partie. Classe V; Mollusques* **1**, 22 (1831).

39. Brandt, R. A. M. The non-marine aquatic mollusca of Thailand. *Archiv für Mollusckenkunde* **105**, 1–423 (1974).

40. van Benthem Jutting, W.S.S. Catalogue of the non-marine Mollusca of Sumatra and of its satellite islands. *Beaufortia* **7**, 41–191 (1959).

41. Bourguignat, J.-R. Histoire malacologique de l'Abyssinie. *Annales des Sciences naturelles (Zoologie et Paléontologie, series 6)* **15**, 1–162 (1883).

42. Brown, D.S. *Freshwater snails of Africa and their medical importance* (London, 1994).

43. Krauss, F. *Die südafrikanischen Mollusken; ein Beitrag zur Kenntniss der Mollusken des Kap- und Natallandes und zur geographischen Verbreitung derselben, mit Beschreibung und Abbildung der neuen Arten* (Stuttgart, 1848).

44. Stothard, J.R., Bremond P., Andriamaro L., Loxton N.J., Sellin B., Sellin E., Rollinson D. Molecular characterization of the freshwater snail *Lymnaea*

natalensis (Gastropoda: Lymnaeidae) on Madagascar with an observation of an unusual polymorphism in ribosomal small subunit genes. *Journal of Zoology* **252**, 303–315 (2000).

45. Lamarck, J.B.P.A. de M. *Histoire naturelle des animaux sans vertèbres. Vol. 6. Pt. 2* (Paris, 1822).

46. Sowerby, G.B. *The genera of recent and fossil shells, for the use of students in conchology and geology* (London, 1822).

47. Subba Rao, N.V. *Handbook of freshwater molluscs of India* (Calcutta, 1989).

48. Glöer, P. & Bössneck, U. Freshwater molluscs from Nepal and North India with the description of seven new species. *Archiv für Molluskenkunde* **142**, 137–156 (2013).

49. Servain, G. *Histoire malacologique du lac Balaton en Hongrie* (Poissy, 1881).

50. Hartmann, J.D.W. System der Erd- und Flußschnecken der Schweiz. *Neue Alpina* **1**, 194–268 (1821).

51. Vinarski, M.V. & Glöer, P. Taxonomical notes on Euro-Siberian freshwater molluscs. 1. *Turbo patulus* Da Costa, 1778 is not a senior synonym of *Limneus ampla* Hartmann, 1821 (Mollusca: Gastropoda: Lymnaeidae). *Ruthenica* **17**, 55–63 (2007).

52. Welter-Schultes, F. *European non-marine molluscs: a guide for species identification* (Göttingen, 2012).

53. Poliński, W. Limnoloshka ispitivanja Balkanskog Poluostrva. I. Reliktna fauna gasteropoda Ochridskog Jezera. *Glas Srpske Kraljevske Akademije* **137**, 129–182 (1929).

54. Albrecht, C., Wolff, C., Glöer, P. & Wilke, T. Concurrent evolution of ancient sister lakes and sister species: the freshwater gastropod genus *Radix* in lakes Ohrid and Prespa. *Hydrobiologia* **615**, 157–167 (2008).

55. Schütt, H. Zwei neue reliktäre Süßwassermollusken der Dinariden. *Annalen des Naturhistorischen Museums in Wien* **78**, 473–480 (1974).

56. Gundrizer, V.A. & Starobogatov, Ya. I. New species of freshwater mollusks of the lower Yenisei basin [In Russian]. *Zoologicheskiy Zhurnal* **58**, 1130-1135 (1979).

57. Geyer, D. *Unsere Land- und Süsswasser-Mollusken* (Stuttgart, 1927).

58. Gundrizer, V.A. Freshwater mollusks of the Middle Siberia and their role in the productivity of waterbodies [In Russian]. in *Biological resources of the inland waterbodies of Siberia and the Far East*. 164–175 (Moscow, 1984).

59. Schrank, F. von P. *Fauna Boica. Durchgedachte Geschichte der in Baiern einheimischen und zahmen Thiere. Bd. 3(2)* (Landshut, 1803).

60. Schniebs, K., Georgiev, D., Glöer, P. & Hundsdoerfer, A. A molecular genetic evidence of the occurrence of the freshwater snail *Radix lagotis* (Schrank,

1803) (Gastropoda, Lymnaeidae) in Bulgaria. *Ecologica Montenegrina* **3**, 29–39 (2015).

61. Studer, S. Kurzes Veszeichniss der bis jetzt in unzern Vaterlande entdecken Conchylien. *Naturwissenschaftlicher Anzeiger der Allgemeinen Schweizerischen Gesellschaft für die Gesammten Naturwissenschaften* **3**, 83–90, 91–194 (1820).

62. Forcart, L. Ipsa Studeri Conchylia. Professor Samuel Studer (1757–1834), seine Bedeutung als Naturforscher und die von ihm hinterlassene Molluskensammlung Mitteilungen der Naturforschenden Gesellschaft in Bern. *Neue Folge* **15**, 157–210 (1957).

63. Kruglov, N.D. & Starobogatov, Ya.I. A contribution to the morphology of European representatives of the subgenus *Peregriana* of the genus *Lymnaea* (Gastropoda, Pulmonata) [In Russian]. *Zoologicheskiy Zhurnal* **62**, 1462-1473 (1983).

64. Schniebs, K., Glöer, P., Vinarski, M. & Hundsdoerfer, A. Intraspecific mophological and genetic variability in *Radix balthica* (Linnaeus 1758) (Gastropoda: Basommatophora: Lymnaeidae) with morphological comparison to other European *Radix* species. *Journal of Conchology* **40**, 657–678 (2011).

65. Müller, O.F. *Vermium terrestrium et fluviatilium seu animalium infusorium, helminthicorum et testaceorum non marinorm succincta historia* (Hafnia et Lipsia, 1774).

66. Falkner, G., Ripken, Th.E.J. & Falkner, M. Mollusques continentaux de France. Liste de référence annotée et bibliographie. *Collection Patrimoines Naturels* **52**, 1–350 (2002).

67. Nekhaev, I.O., Shiøtte, T. & Vinarski, M.V. Type materials of European freshwater molluscs described by Otto Friedrich Müller. *Archiv für Molluskenkunde* **144**, 51–64 (2015).

68. Schniebs, K., Glöer, P., Vinarski, M. & Hundsdoerfer, A. Intraspecific morphological and genetic variability in the European freshwater snail *Radix labiata* (Rossmaessler, 1835) (Gastropoda: Basommatophora: Lymnaeidae). *Contributions to Zoology* **82**, 55−68 (2013).

69. Vinarski, M.V., Aksenova, O.V., Bespalaya, Yu.V., Bolotov, I.N., Schniebs, K., Gofarov, M.Yu. & Kondakov, A.V. *Radix dolgini*: The integrative taxonomic approach supports the species status of a Siberian endemic snail (Mollusca, Gastropoda, Lymnaeidae). *Comptes Rendus Biologies* **339**, 24–36; DOI:10.1016/j.crvi.2015.11.002 (2016).

70. Vinarski, M.V. Recent species name changes in the European Lymnaeidae: two tales with unhappy end? *Ruthenica* **27**, 141–153 (2017).

71. Kruglov, N.D. & Starobogatov, Ya.I. A contribution to the morphology and taxonomy of the subgenus *Peregriana* (*Lymnaea,* Gastropoda, Pulmonata)

of the Asiatic part of the USSR and adjacent regions [In Russian]. *Zoologicheskiy Zhurnal* **63**, 22-33 (1984).

72. Middendorff, A.T. Mollusken. in Middendorff, A.T. *Reise in den äussersten Norden und Osten Sibiriens. Band II. Zoologie. Theil 1. Wirbellose Thiere*. 163–464 (Saint Petersburg, 1851).

73. Aksenova, O.V., Vinarski, M.V., Bolotov, I.N., Beslapaya, Yu.V., Kondakov, A.V. & Paltser, I.S. An overview of *Radix* species of the Kamchatka Peninsula (Gastropoda: Lymnaeidae). *The Bulletin of the Russian Far East Malacological Society* **20**, 5–27 (2016).

74. Reeve, L.A. On a new species of *Lymnaea* from Tibet. *Proceedings of the Zoological Society of London* **18**, 49 (1850).

75. Kobelt, W. *Illustrirtes Conchylienbuch* (Nürnberg, 1881).

76. Boettger, O. Die Binnenmollusken Transkaspiens und Chorassans. *Zoologische Jahrbücher. Abteilung für Systematik, Geographie und Biologie der Tiere* **4**, 925–992 (1889).

77. Kruglov, N.D. & Starobogatov, Ya.I. 1985. The volume of the subgenus *Galba* and of other similar subgenera of the genus *Lymnaea* (Gastropoda, Pulmonata) [In Russian]. *Zoologicheskiy Zhurnal* **64**, 24–35.

78. Ponder, W.F. & Waterhouse, J.H. A new genus and species of Lymnaeidae from the lower Franklin river, south western Tasmania, Australia. *Journal of Molluscan Studies* **63**, 441–448 ; DOI:10.1093/mollus/63.3.441 (1997).

79. Schniebs, K. et al. A new alien species in Europe: First record of *Austropeplea viridis* (Quoy & Gaimard, 1833) in Spain. *Journal of Conchology* **42**, 357–370 (2017).

80. Quoy, J.R.C. & Gaimard, J.P. *Voyage de découvertes de l'"Astrolabe" exécuté par ordre du Roi, pendant les années 1826-1829, sous le commandement de M. J. Dumont d'Urville. Zoologie* (Paris, 1832).

81. Gould, A.A. Descriptions of shells collected in the North Pacific Exploring Expedition under Captains Ringgold and Rodgers. *Proceedings of the Boston Society for Natural History* **6**, 422-426; **7**, 40-45, 138-142, 161-166 (1859).

82. Johnson, R.I. The Recent Mollusca of Augustus Addison Gould. *United States National Museum Bulletin* **239**, 1–182 (1964).

83. Preston, H.B. Report on a small collection of freshwater Mollusca (*Limnaea* and *Pisidium*) from Tibet. *Records of Indian Museum* **3**, 115–116 (1909).

84. Bergh, R. Bullacea. *Reisen in Archipel der Philippinen von Dr. C. Semper* **7**, 209–256 (1901).

85. Pfeiffer, L. Description of a new species of *Amphipeplea*. *Transactions of the Zoological Society of London* **13**, 68 (1845).

86. Vinarski, M.V. Annotated type catalogue of lymnaeid snails (Mollusca, Gastropoda) in the collection of the Natural History Museum, Berlin. *Zoosystematics & Evolution* **92**, 131–152; DOI:10.3897/zse.92.8107 (2016).
