# Supplementary material for: Species Richness, Molecular Taxonomy and Biogeography of the Radicine Pond Snails (Gastropoda: Lymnaeidae) in the Old World
Source: Sci Rep. 2018 Jul 25;8:11199. doi: 10.1038/s41598-018-29451-1 (PMC6060155; doi:10.1038/s41598-018-29451-1)
Supplement: Supplementary file 3 — Supplementary Dataset 2 [file 41598_2018_29451_MOESM3_ESM.docx]

**Supplementary Dataset 2.** National checklists of the radicine pond snails (Lymnaeidae: Amphipepleinae) for countries and regions of the Old World based on the available COI sequences (1 January 2018)

**Albania**

*Ampullaceana relicta*

*Ampullaceana* sp. Ohrid

*Peregriana peregra*

*Radix auricularia*

**Armenia**

*Radix auricularia*

**Austria**

*Ampullaceana ampla*

*Ampullaceana balthica*

**Bulgaria**

*Ampullaceana fontinalis*

**China**

*Radix auricularia*

*Radix* *makhrovi* **sp. nov.**

*Radix* *brevicauda*

*Radix* *plicatula*

*Tibetoradix hookeri* **gen. et comb. nov.**

*Tibetoradix* *kozlovi* **sp. nov.**

*Tibetoradix* sp.1

*Tibetoradix* sp.2

*Tibetoradix* sp.3

*Tibetoradix* sp.4

*Orientogalba ollula*

*Orientogalba* cf. *bowelli*

**Croatia**

*Peregriana peregra*

*Radix auricularia*

**Finland**

*Myxas glutinosa*

**France**

*Ampullaceana balthica*

*Ampullaceana intermedia*

*Peregriana peregra*

*Radix auricularia*

**France:** **Réunion Island, Indian Ocean**

*Radix rubiginosa*

**Germany**

*Ampullaceana ampla*

*Ampullaceana balthica*

*Ampullaceana fontinalis*

*Peregriana peregra*

*Radix auricularia*

**Great Britain**

*Ampullaceana balthica*

*Radix auricularia*

**Greece**

*Ampullaceana lagotis*

*Peregriana peregra*

*Radix auricularia*

*Radix* sp. Trichonis

**Hungary**

*Ampullaceana fontinalis*

**Iceland**

*Ampullaceana balthica*

**India**

*Cerasina luteola*

*Radix* *rufescens*

**Indonesia**

*Radix rubiginosa*

*Orientogalba* cf. *viridis*

**Iraq**

*Radix* *euphratica*

**Italy**

*Peregriana peregra*

*Radix auricularia*

**Kyrgyzstan**

*Radix auricularia*

**Latvia**

*Ampullaceana balthica*

**Macedonia**

*Ampullaceana* *relicta*

*Ampullaceana* sp. Ohrid

*Peregriana peregra*

*Radix auricularia*

**Montenegro**

*Ampullaceana ampla*

*Peregriana peregra*

*Radix auricularia*

**Myanmar**

*Cerasina* *siamensis*

*Radix* *rufescens*

**Nepal**

*Cerasina luteola*

*Cerasina oxiana*

*Radix* *rufescens*

*Radix alticola*

*Orientogalba ollula*

**Poland**

*Ampullaceana ampla*

*Ampullaceana balthica*

*Ampullaceana fontinalis*

*Ampullaceana* cf. *dipkunensis*

*Radix* *auricularia*

**Romania**

*Radix auricularia*

**Russia**

*Ampullaceana balthica*

*Ampullaceana fontinalis*

*Ampullaceana lagotis*

*Ampullaceana* cf. *dipkunensis*

*Kamtschaticana kamtschatica*

*Peregriana dolgini*

*Peregriana peregra*

*Radix auricularia*

*Radix* *euphratica*

**Serbia**

*Peregriana peregra*

**Singapore**

*Radix rubiginosa*

**Slovakia**

*Peregriana peregra*

**Slovenia**

*Peregriana peregra*

**South Korea**

*Orientogalba ollula*

**Spain**

*Ampullaceana balthica*

*Ampullaceana intermedia*

*Radix auricularia*

**Sweden**

*Ampullaceana balthica*

**Switzerland**

*Ampullaceana ampla*

*Ampullaceana balthica*

*Ampullaceana fontinalis*

*Peregriana peregra*

*Radix auricularia*

**Tajikistan**

*Ampullaceana lagotis*

*Cerasina oxiana*

*Radix alticola*

*Radix auricularia*

*Radix* *euphratica*

**Thailand**

*Cerasina* *siamensis*

*Radix rubiginosa*

**Turkey**

*Ampullaceana fontinalis*

*Peregriana peregra*

**Vietnam**

*Radix* *plicatula*

**USA (Alaska)**

*Radix auricularia*

**Africa: Cabo Verde**

*Radix natalensis*

**Africa: Egypt**

*Radix natalensis*

**Africa: Malawi**

*Radix natalensis*
